# Synergistic Effects in Matrix-Embedded Alloy Nanoclusters: Advanced Type‑I Photosensitizers for Theranostics

**DOI:** 10.1021/acsami.5c22942

**Published:** 2026-01-28

**Authors:** Negar Hosseiniyan, Pietro Castronovo, Gregory Beaune, Eslam Abdelrady, Xi Chen, Artem Zhyvolozhnyi, Hamza Siddiqui, Jahan Farhana, Hua Jiang, Minna Makki, Marco Cannas, Alice Sciortino, Ilya Skovorodkin, Anatoliy Samoylenko, Seppo J. Vainio, Fabrizio Messina, Sourov Chandra

**Affiliations:** † Department of Applied Physics, 174277 Aalto University, P.O. Box 15100, FI-00076 Espoo, Finland; ‡ Dipartimento di Fisica e Chimica - Emilio Segrè, Università degli Studi di Palermo, Via Archirafi 36, 90123 Palermo, Italy; § ATeN Center, Università degli studi di Palermo, viale delle scienze, Edificio 18, 90128 Palermo, Italy; ∥ Laboratory of Developmental Biology, Disease Networks Research Unit, Faculty of Biochemistry and Molecular Medicine, Infotech Oulu, Kvantum Institute, Oulu University, Aapistie 5A, 90014 Oulu, Finland; ⊥ iCell Group, Research and Development, Finnish Red Cross Blood Service, Haartmaninkatu 8, FIN-00290 Helsinki, Finland

**Keywords:** gold nanoclusters, cellulose nanocrystals, photosensitizer, ROS, theranostics

## Abstract

A combination of biomedical imaging and photodynamic therapy (PDT) in a single nanomaterial would be a breakthrough in nanomedicine. However, devising a single photosensitizer capable of efficient PDT without requiring an external oxygen source under typically hypoxic tumor conditions, combined with high photostability, biocompatibility, and renal clearance, remains a challenge. Atomically precise ultrasmall (<2 nm) gold nanoclusters (AuNCs) are emerging as potential multifunctional biomedicines, encompassing imaging, diagnosis, and therapy in a single nanoplatform. Herein, we report bioderived cellulose nanocrystal-supported gold nanoclusters (CNC-AuNCs) with selective mono or multiheteroatom (Ag, Pd, and Pt) substitution at the core of the nanoclusters. The replacement of one or more gold atoms significantly modulates their emission wavelengths, photoluminescence quantum yields, as well as excited-state relaxation kinetics. These materials can easily penetrate the cells, accumulating in the cytoplasm and emitting bright luminescence. While the nanocomposites are highly biocompatible, they can produce reactive oxygen species (ROS) through the formation of free radicals (O_2_^–·^ and ^·^OH) upon exposure of light. The synergistic effect of the light absorption by the matrix and the diverse excited-state relaxation pathways of the nanoclusters results in the efficient generation of ROS in variable concentrations, ultimately leading to the complete destruction of targeted cancer cells via Type-I photodynamic effect. The optimal ROS efficacy combined with minimal cytotoxicity suggests a universal strategy for developing strong PDT-I agents, paving the way for versatile nanomaterials in theranostic applications.

## Graphical abstract

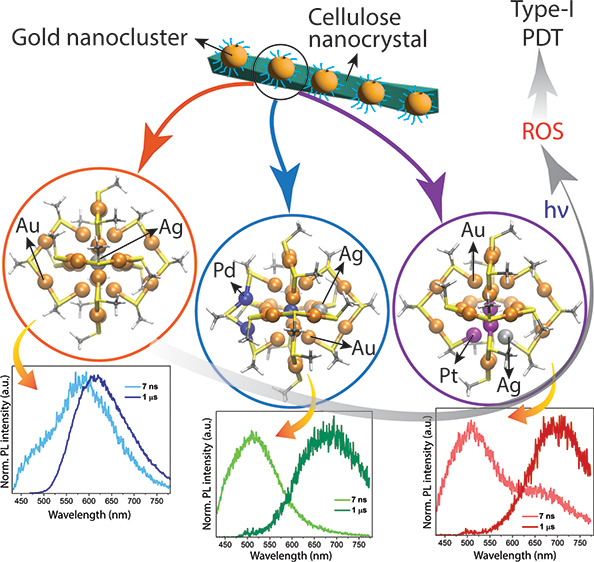

## 1 Introduction

Photodynamic therapy (PDT) is an emerging, non- or minimally invasive therapeutic approach that has gained significant attention in the field of medical science for the treatment of various diseases, including cancers.^1,2^ PDT involves the administration of a photosensitive agent, known as a photosensitizer (PS), which is targeted to the tumor tissues.^3^ Upon exposure to specific wavelengths of light, the PS generates reactive molecular species, such as singlet oxygen or free radicals, leading to localized cell damage and eventual destruction of the targeted cells.^1,4,5^ Although PDTs offer several advantages, including being minimally invasive, selectively targeting, causing minimal scarring, and reducing systemic side effects, their use has so far remained primarily limited to the treatment of skin cancers, such as actinic keratoses and some early stage nonmelanoma skin cancers.^6,7^ Drawbacks include limited tissue penetration of light, photosensitivity, poor biodegradability, cytotoxicity or side effects from photosensitizing drugs, the prerequisite for an external source of oxygen, accumulation in the liver and kidneys after treatments, and effectiveness limited to skin conditions.^1,8^ The most widespread organic PSs used in PDT, consisting of porphyrin-based molecular systems, exhibit limited depth of effectiveness, poor selectivity, hydrophobicity issues, and high photosensitivity. For example, Chlorin e6 (Ce6) is one of the FDA-approved organic PSs that demonstrates the required clinical properties for PDT.^9^ However, in addition to its poor solubility and limited tissue penetration, Ce6 could also be responsible for localized photosensitive reactions, potential damage to surrounding tissues, pain, and inflammation, as well as possible risks of burns, blistering, and allergic reactions.^3,9,10^ On the other hand, inorganic PSs, such as TiO_2_ and ZnO nanoparticles or semiconductor quantum dots, show poor biocompatibility along with potential long-term toxicity and side effects associated with their persistent accumulation in specific tissues and organs.^1,11–13^ Therefore, ensuring renal clearance after treatment is essential to prevent accumulation and adverse effects.^14–16^

In recent years, the combination of nanotechnology and biomedical imaging has advanced significantly in the field of medical science, especially in nanotheranostics, that is, the use of a single nanosystem for combined diagnostics and targeted therapy. Among these innovations, gold nanoclusters (AuNCs) have emerged as versatile agents with potential in theranostics.^17,18^ AuNCs are ultrasmall assemblies of gold atoms, typically 1–2 nm in diameter.^19,20^ These nanoclusters (NCs) possess high surface area, excellent biocompatibility, and tunable optical properties due to their ultrasmall size and well-defined structure, making them ideal candidates for imaging and PDT.^20–24^ In addition, easy renal clearance further promotes AuNCs as potentially suitable systems for noninvasive imaging and therapeutic purposes while minimizing tissue accumulation and long-term toxicity.^14^ However, their poor photoluminescence (PL) quantum yields (QYs), compared with those of quantum dots or organic PSs, restrict their potential applications. Huang et al. have described the successful development of Ce6-conjugated silica-coated AuNCs with a phototheranostic formula that offers higher cellular uptake and fluorescence imaging-guided enhancement of PDT.^25^ Similarly, Zhang et al. have reported an AuNC-based Ce6 delivery nanoplatform for targeted PDT, in which the nanoprobes exhibit rapid Ce6 release within hours.^26^ Vankayala et al. have shown a distinctive multifunctional TAT peptide (peptide sequence: N-GRKKRRQRRR-C)-conjugated AuNC-based theranostic nanoplatform designed for nuclear targeting, enabling simultaneous fluorescence imaging, gene delivery, and long-wavelength near-IR light-activated photodynamic cancer therapy.^27^ Finally, Han et al. have shown that lipoic acid-functionalized AuNCs could produce a strong two-photon photodynamic (PD) effect without requiring any external source of oxygen (Type-I PDT).^28^

In an active PS, not only the PL QY, but also the detailed nanosecond (ns) and subnanosecond relaxation cascade from singlet (S1) and triplet (T1) excited states to ground state impacts PD effects.^29,30^ Such excited-state relaxation pathways could influence energy deposition in living tissues and control the ability of the PS to convert absorbed light into cytotoxic reactive molecular species. The optical population of the T1 state, and the subsequent molecular relaxation from it, can transfer electrons to cellular oxygen or water molecules, ultimately leading to the generation of singlet oxygen and free radicals.^29^ As a result, the relaxation dynamics, electron population, lifetime, and excited-state relaxation pathways of AuNCs are the key factors controlling the precision, efficiency, and selectivity of Type-I PDT.

The incorporation of certain heteroatoms in specific sites within the AuNCs provides a route to control their optical and electronic properties, PL QYs, and relaxation dynamics, by tuning their absorption and emission transitions.^31–34^ Indeed, the type, concentration, and distribution of dopant elements in the Au core of the NCs can have a significant impact on their optical properties while also preventing aggregation and minimizing surface defects, ultimately leading to increased resistance against degradation or photobleaching.^31,34^ Although the incorporation of heteroatoms significantly alters the optical and electronic properties responsible for the catalytic activity of AuNCs, their influence on PDT is still largely unknown. Moreover, tightly controlled chemical strategies for heteroatom doping are paramount, as both the concentration of dopants and the size of the resultant NCs play crucial roles in restraining their cytotoxicity.^35^

Herein, we demonstrate a new strategy to achieve well-controlled and position-dependent mono- and multimetal substitution of Ag, Pt, and Pd on matrix-supported AuNCs functionalized by glutathione. Steady-state and time-resolved optical spectra, along with DFT calculations, are used to fully model the overall photocycles of these nanosystems. The specific positioning and number of heteroatoms in the core of the AuNCs influence the optical responses and excited-state relaxation pathways. As a result, the cellulose nanocrystal-supported nanoclusters (CNC-NCs) show strong photodynamic effects while being nontoxic. The results are very promising in view of the development of AuNCs as a new family of nanomedicines for simultaneous imaging and PDT.

## 2 Results and Discussion

### 2.1 Synthesis and Characterizations

Our strategy enables the controlled assimilation of mono- and bimetallic dopants into the core of gold nanoclusters, producing PL with excited-state relaxation dynamics that differ significantly from those of the parent NCs. Details of the synthesis are provided in Section 4. Notably, the substitution of Au atoms in the core of AuNCs with mono- and bimetallic heteroatoms is made possible by the assistance of −OSO_3_^–^ groups over the surfaces of cellulose nanocrystals (CNCs), providing a supporting matrix.^36^ Through this approach, we achieve precise control with minimal number of dopant elements in AuNCs, allowing the development of a range of Ag-, Pd-, and Pt-doped or codoped AuNCs with enhanced and tunable optical properties. The whole process is summarized in Figure 1. While in the absence of CNCs, the introduction of AgNO_3_ into an aqueous solution of HAuCl_4_ or H_2_PtCl_6_ results in rapid aggregation; however, preadsorption of metal ions onto CNCs stabilizes the precursors via −OSO_3_^–^ groups.

**Figure 1:**
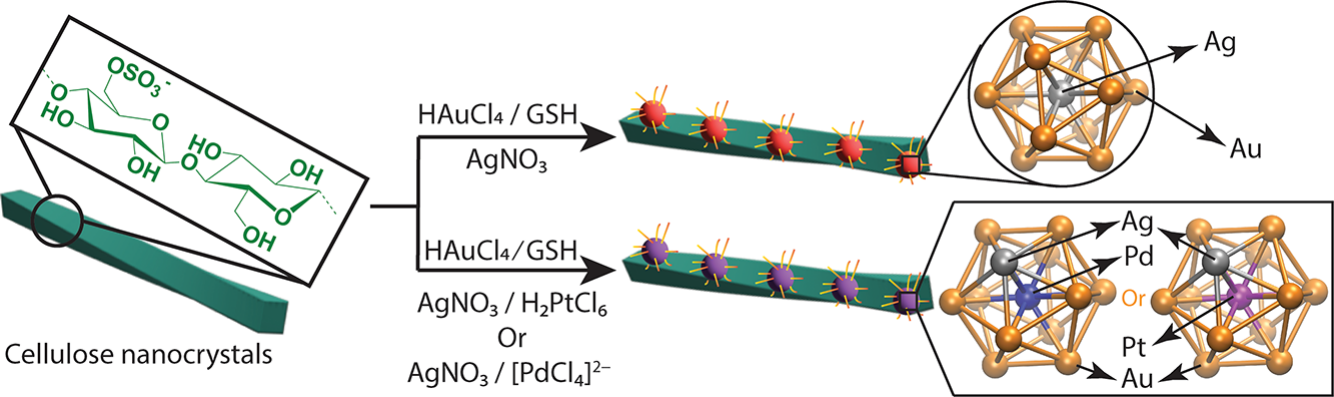
Schematic representation of the preparation of Ag-doped and Ag/Pt or Ag/Pd codoped AuNCs over the surfaces of cellulose nanocrystals (CNCs).

To confirm the composition of the nanoclusters over CNC surfaces, atomic absorption spectroscopy (AAS) as well as inductively coupled plasma mass spectrometry (ICP-MS) were performed. The results confirm the success of the procedure and show that all of the dopants are present only in trace amount relative to the gold (Tables S1 and S2, Supporting Information), i.e., from one to three dopant atoms per cluster. The STEM images in Figure 2a–f reveal that all samples present a quite similar morphology.^36,37^ The particles, ca. 1.3 nm in size, are grafted over the CNC surfaces regardless of the dopant elements. Dynamic light scattering further confirms that the heteroatom doping does not significantly affect the size or stability of these nanocomposites (Table S3, Supporting Information). This implies that the addition of dopant atoms to AuNCs does not significantly alter their overall size, structure, or the pattern of nanoclusters over CNCs. While AuNCs exhibit a nearly neutral zeta potential at acidic and neutral pH, they become strongly negative at alkaline pH due to deprotonation of the carboxylic acid group in glutathione (Table S3, Supporting Information). On the contrary, CNCs consistently maintain high negative zeta potentials across pH ranges, providing a stable matrix for AuNCs without disrupting their surface charge (Table S3, Supporting Information). Additional information is obtained from X-ray photoelectron spectroscopy (XPS). Au 4f (Figure S1a, Supporting Information) and C 1s (Figure S1b, Supporting Information) XPS spectra reveal that the surface functionalities as well as the oxidation states of Au in all of the samples with mono- or multimetal doping are quite similar. In addition, S 2p spectra (Figure S1c, Supporting Information) again confirm the presence of both −OSO_3_^–^ groups over the CNC surfaces and the Au–S bonds in these clusters.^36,37^ In conclusion, no major discrepancies have been observed in the morphologies and surface functionalities of CNCs and AuNCs in the absence and presence of different heteroatoms. Finally, Pd 3d, Pt 4f, and Ag 3d XPS spectra (Figure 2g–i) validate the presence, within the NCs, of trace amounts of Pd, Pt, and Ag atoms, respectively. Interestingly, while all Pt atoms in these NCs remain in the Pt (0) oxidation state, Pd atoms exhibit mixed valence states, Pd (II) (major) and Pd (0) (minor), in all of the Pd-doped samples.

**Figure 2:**
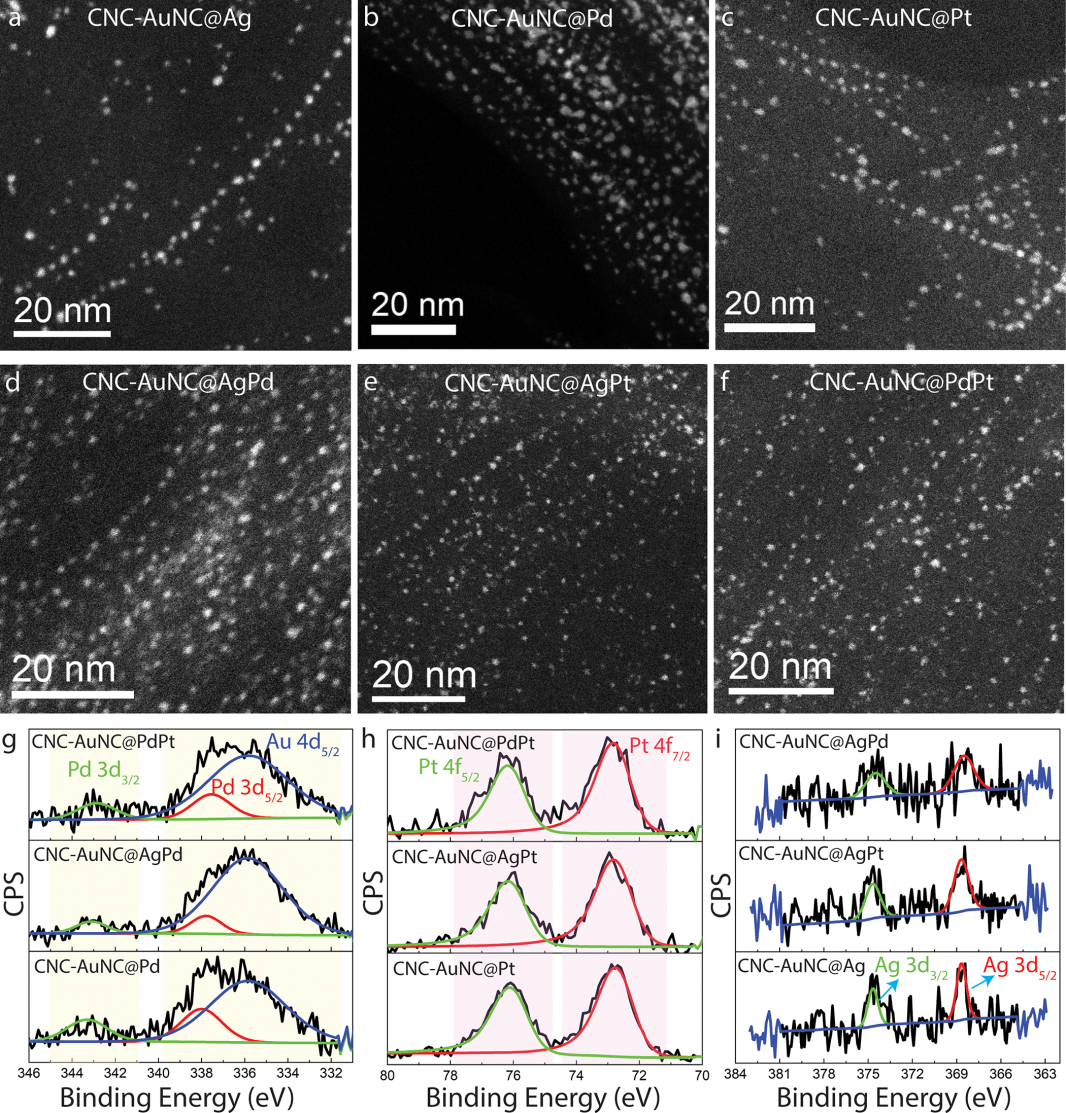
(a–f) STEM images and (g) Pd 3d, (h) Pt 4f, and (i) Ag 3d XPS spectra of different heteroatom-doped CNC-NCs.

### 2.2 Optical Properties

To investigate the effect of heteroatoms on the photophysical properties of AuNCs, the aqueous dispersions of CNC-NCs have been analyzed by steady-state, nanosecond (ns), and femtosecond (fs)-resolved spectroscopic measurements. The UV–vis spectra (Figure S2, Supporting Information) show featureless absorption profiles from 300 to 800 nm, appearing to be dominated by scattering from the CNCs. Photoluminescence excitation (PLE) spectra of the CNC-NCs at their PL maxima, being unaffected by scattering, allow one to elucidate the effect of the dopants on their optical absorption (Figure S3, Supporting Information). Usually, the substitution of Au by Ag atoms into gold nanoclusters causes blue shifts in their absorption bands.^31,38,39^ A similar trend has also been noticed after the introduction of Ag atoms in CNC-AuNCs. We observe that the PLE peak maximum blueshifts by about 40 nm upon going from CNC-AuNC to CNC-AuNC@Ag. On the contrary, two absorption peaks have been observed in CNC-AuNC@AgPd, one at the position already seen for CNC-AuNC@Ag (339 nm) and an additional red-shifted one centered at 398 nm.^40^ CNC-AuNC@AgPt shows a similar PLE spectrum with respect to CNC-AuNC@AgPd; however, the secondary peak at 398 nm is much less prominent in this case. Each sample presents a distinctive steady-state PL spectrum (Figure 3a), with a peak position ranging from 600 to 700 nm. Despite the degree of control of the atomic structures of the alloy NCs, there are boundary conditions imposed by the chemical properties of the dopants and the stability of the structure. The peak patterns are quite different from each other and depend solely on the dopant elements. The absolute quantum yields (QYs), listed in Table S5 (Supporting Information), reveal that Ag doping results in a substantial rise in PL efficiency (from 3.5 to 23% PL QY), while Pd or Pt doping implies a sharp decrease of the PL QY (1.9 and <0.1% QY, respectively). Moreover, a significant drop down of the PL QYs from CNC-AuNC@Ag (QY = 23%) to CNC-AuNC@AgPd (QY = 2.9%) or CNC-AuNC@AgPt (QY = 4.4%) has been observed by introducing either Pd or Pt atoms in Ag-doped AuNCs, with the resulting NCs having QYs quite comparable to that of the undoped CNC-AuNCs (QY = 3.5%). We chose [Au_25_(SR)_18_]^−1^ (SR = glutathione) as a theoretical model, not an experimentally confirmed structure, to understand how doping affects the optical properties and excited-state dynamics in AuNCs. The observed trends in PL QYs can be elucidated by DFT calculations (Figures S4 and S5, Supporting Information), showing that accumulation of Pt and Pd atoms in the clusters results in a HOMO–LUMO gap smaller than that of both the undoped and Ag-doped AuNCs. On the other hand, further codoping with one additional Ag atom in the outer core of the NCs has a minor effect on their HOMO–LUMO gap (Table S4, Supporting Information). Because the substantial QY enhancement has been well established as a fingerprint of successful Ag doping in the central position,^36,41^ the absence of a comparable enhancement in the bimetallic AgPt- and AgPd-doped NCs strongly suggests that the Pd or Pt dopants compete with Ag in occupying the central position of the nanocluster.^42^

**Figure 3:**
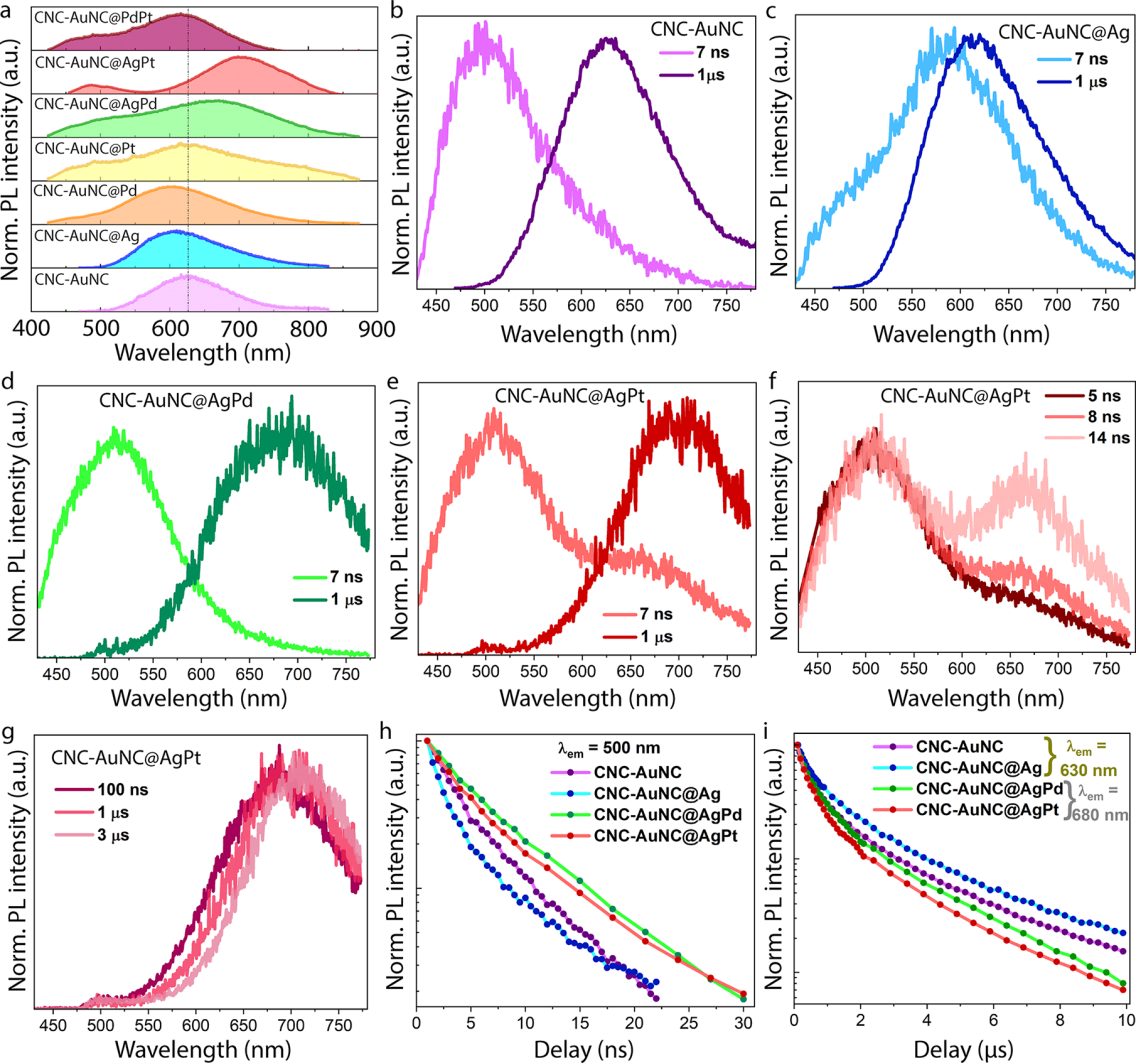
(a) Normalized steady-state PL spectra of undoped and monometal- and bimetal- doped CNC-AuNCs, excited at 410 nm. Corresponding time-resolved PL spectra of (b) CNC-AuNC, (c) CNC-AuNC@Ag, (d) CNC-AuNC@AgPd, and (e) CNC-AuNC@AgPt at 7 ns and 1 μs delays, highlighting the presence of the green and orange/red bands. (f, g) PL spectra of CNC-AuNC@AgPt at relevant delays, highlighting (f) rise and (g) spectral shift of the red band. (h,i) Decimal semilogarithmic plots of the green (h) and orange/red (i) band decays.

Time-resolved PL measurements show that the broad steady-state PL spectrum arises from the composition of a green (G) and an orange/red (O/R) band, with lifetimes in the nanosecond and μs range, respectively (Figure 3b–g). Interestingly, a gradual rise of the O/R band concurrently with the nanosecond decay of the G-band was also observed, as exemplified in CNC-AuNC@AgPt in Figure 3f. Such a result endorses the idea that the state responsible for the O/R band emission (O/R state) is populated from the one determining the G-band emission (G state). Furthermore, the long decay lifetimes of the O/R band suggest that the O/R state has a strong triplet character, and, consequently, that the process responsible for its initial population from the G-state manifold (discontinuous arrow in Figure 4e) is an intersystem crossing (ISC).^43,44^ Finally, a red shift was observed during the decay of the R–O band (Figure 3g). The peak wavelength and relative intensity of the two components depend upon the specific sample, as reported in Table S6 (Supporting Information). It is worth noting that the O/R component is always dominant. In particular, nanosecond-resolved PL measurements reveal that both the green and the orange/red emissions (Figure 3h,i) decay biexponentially. The former shows lifetimes of τ_1_ ranging from ∼1 to ∼5 ns and τ_2_ ∼ (7 to 16) ns (Figure S6 and Table S7, Supporting Information), whereas the latter shows lifetimes of τ_1_ ∼ 500 to 900 ns and τ_2_ ∼ 2 to 3 μs (Figure S7 and Table S8, Supporting Information).

**Figure 4:**
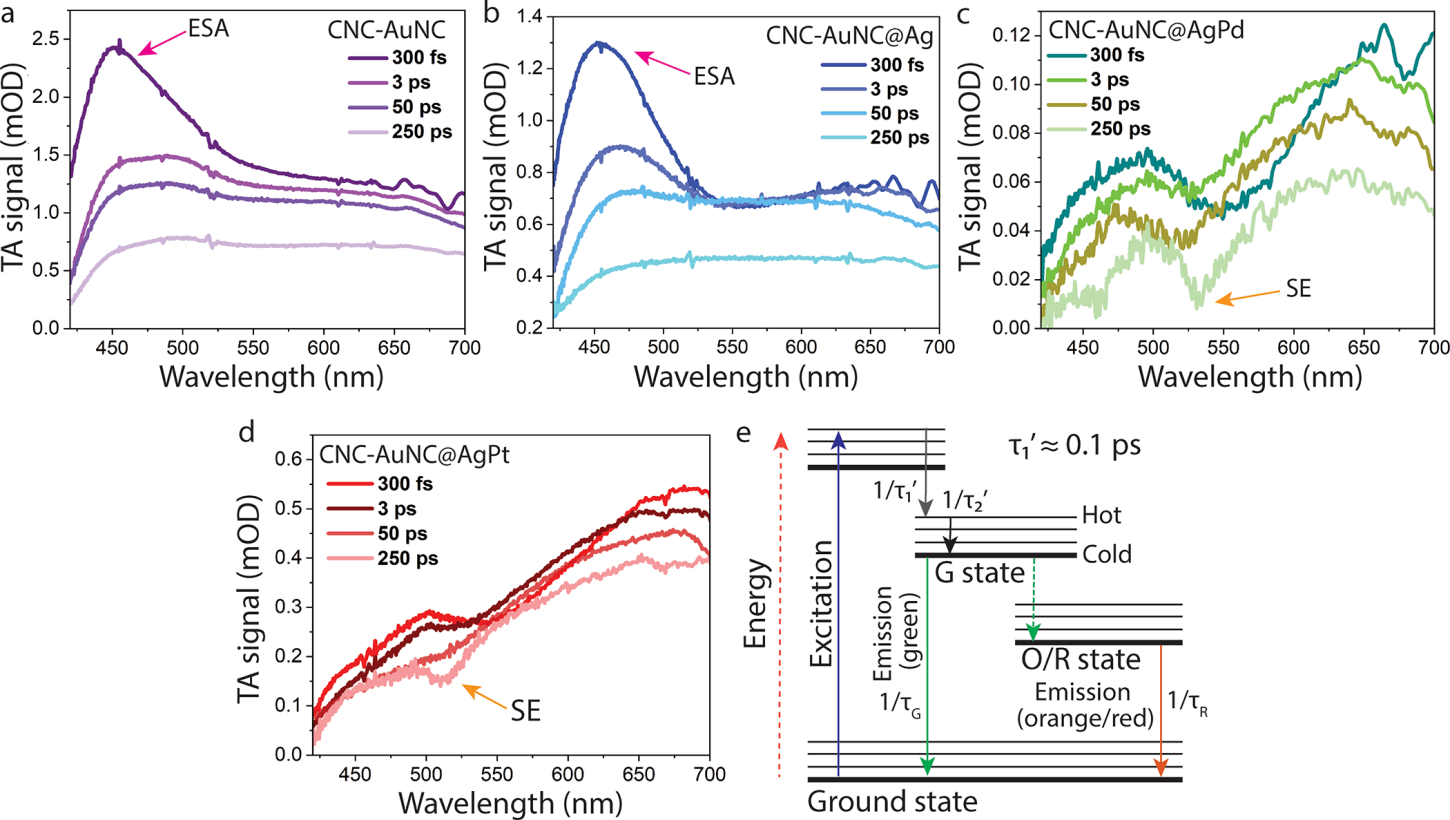
Transient absorption spectra of (a) CNC-AuNC, (b) CNC-AuNC@Ag, (c) CNC-AuNC@AgPd, and (d) CNC-AuNC@AgPt (pumped at 400 nm) at several pump–probe delays. (e) Schematic representation of the photocycle, highlighting the main transitions with time scales of the relevant processes, as obtained by fitting the kinetic traces obtained from TA and nanosecond-resolved fluorescence data. Table 1

Finally, femtosecond-resolved transient absorption (TA) measurements have been performed to investigate the subnanosecond dynamics of the CNC-NCs (Figure 4a–d). Notably, the spectra are dominated by a broad and scarcely structured positive excited-state absorption (ESA) signal covering the whole spectral range. Such a broad ESA signal arises from several unresolved transitions from the initial excited state to a large variety of accessible higher excited states, while the stronger TA signal in CNC-AuNC and CNC-AuNC@Ag indicates larger extinction coefficients of the associated transitions. For CNC-AuNC and CNC-AuNC@Ag, the ESA signal shows a defined peak around 450 nm, which disappears anyway within a few picoseconds (ps) as a consequence of an internal conversion (IC) from the initially excited states down to the emissive ones.^36^ On the other hand, CNC-AuNC@AgPd and CNC-AuNC@AgPt exhibit a downward dip in the spectra, resulting from the superposition between the positive ESA envelope and a narrower, negative signal. As the spectral position of the dip matches that of the G-band emission, this signal can be interpreted as stimulated emission (SE), whereby the interaction between the probe pulse and the photoexcited system causes the decay of the latter to the ground state and the contextual emission of a photon, resulting in a negative differential absorption signal.^45^ The observation of SE features can be seen as a way to detect PL from a transient absorption measurement. As shown in Figure 4c,d, this component is visible only at long pump–probe delays, corresponding to the downward dip at 520–530 nm in the spectra at 250 ps. The slow rise of such a signal clearly indicates that the G state is not populated instantaneously. In contrast, relaxation processes such as the above-mentioned IC from higher energy states within the G manifold drive the system from the initially excited high energy states to the G state. The apparent lack of a comparable SE signal in the cases of CNC-AuNC and CNC-AuNC@Ag, in spite of these two samples also displaying a G-band, is most likely due to the SE contribution being buried under the very intense ESA signals. The overall shape of the TA signal in CNC-AuNC and CNC-AuNC@Ag, especially at short times, is very different from that in CNC-AuNC@AgPd and CNC-AuNC@AgPt. The differences provide experimental evidence of the radically different PDOS in monometal- doped versus bimetal doped CNCs, as predicted by DFT calculations (Figures S4 and S5, Supporting Information). However, the different doping appears to influence much more the upper excited states than the first excited state from which steady-state emission is obtained. In fact, TA spectral shapes undergo quite dramatic changes from sample to sample, the differences being much more pronounced than those observed among the steady-state emission spectra (Figure 3a).

Kinetic traces at relevant wavelengths are extracted from the TA signal and fitted by a multiexponential decay model function (Figure S8, Supporting Information). As reported in Table S9 (Supporting Information), the subnanosecond relaxation dynamics of all samples are essentially described by two time scales τ_1_′ and τ_2_′. While the former is always on the order of 0.1 ps, the latter strongly changes from sample to sample. The shorter time scale can be interpreted as an IC from the initial excited state to the G state, while the longer corresponds to a slower relaxation within the G-state manifold (Figure 4e). Only after this τ_2_′ relaxation is the steady state emitting state finally populated, as inferred from the delayed appearance of the SE signal.

The information obtained from steady-state, nanosecond, and femtosecond-resolved measurements allows us to devise a comprehensive picture of the photocycle, as schematized in Figure 4e. Following the initial photoexcitation, the system initially experiences an IC (rate = 1/τ_1_′) into the G-state manifold. The system then undergoes cooling, transitioning from “hot” to “cold” states within the emitting G manifold, with kinetics (rate = 1/τ_2_′) that strongly depend on the specific sample. From the “cold” G state, the system either returns to the ground state, resulting in the G-band emission, or undergoes an intersystem crossing transition to the O/R state, characterized by substantial triplet character. The measured rate 1/τ_G_ is the combined rate of these two processes. From the O/R state, the system finally reaches the ground state, producing the O/R emission (rate = 1/τ_R_). The values of these time scales are summarized in the table embedded in Figure 4. Interestingly, the values of τ_R_ are almost identical from AuNC to AuNC@Ag, but then decrease more than 2-fold in the order AuNC@Ag > AuNC@AgPd > AuNC@AgPt (Table 1). Therefore, the permanence time in the triplet state significantly depends on the doping patterns. Moreover, τ_2_′ strongly increases from undoped CNC-AuNC to its doped counterparts. Therefore, another important effect of doping heteroatoms on the photocycle is to significantly slow down the cooling process within the G-state manifold.

### 2.3 ROS Generation and Type-I Photodynamic Effect

CNC-NCs are found to produce effective ROS upon exposure to light. To prove this phenomenon, we have tested ROS production in 786-O human renal cell carcinoma cells by using a DCFH-DA assay-specific probe.^21,23,28^ The exposure of cells preincubated with CNC-NCs to blue light (λ_ex_ = 405 nm) for 30 min led to a strong increase in the ROS levels as compared to the control (unexposed) cells (Figure 5a). Among the doped clusters, the highest level of ROS induction has been observed for CNC-AuNC@Ag and CNC-AuNC@AgPd composites, whereas it is lowest for CNC-AuNC@AgPt. The intracellular ROS production has been further confirmed by confocal microscopy, using the DCFH-DA assay. In this regard, the 786–0 cells are exposed to blue light (405 nm, 310 mW) for 30 min in the presence of CNC-AuNC@Ag (Figure S9, Supporting Information). A strong increase in green fluorescence was observed in the cells treated with CNC-AuNC@Ag and exposed to blue light, compared to the control cells, confirming intracellular ROS production upon light exposure. To evaluate the mechanistic pathways of such ROS generations, electron spin resonance (ESR) was performed. In this context, the 5,5-dimethyl-1-pyrroline*n*-oxide (DMPO)-based spin-trapping technique was executed to determine the formation of superoxide anions (O_2_^–·^) and hydroxyl radicals (^·^OH) under the exposure of light.^28^ It is observed that CNC-NCs can produce DMPO–OH spin adducts under light irradiation, confirming that ROS generation is significantly dependent on the formation of O_2_^–·^ or ^·^OH radicals, i.e., Type-I PD effect (Figure 5b).

**Figure 5:**
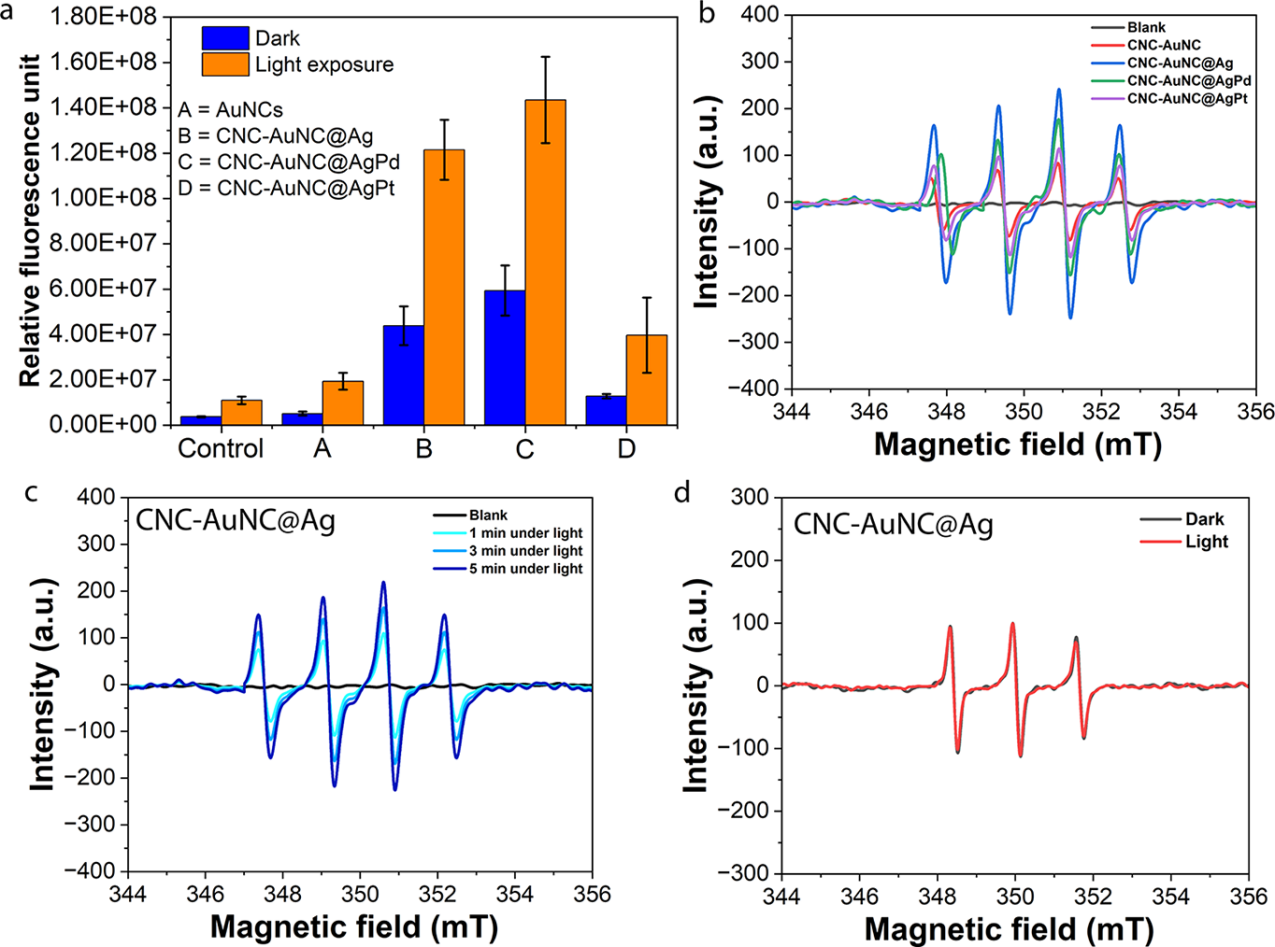
(a) ROS production by different CNC-NCs in 786-O cells using DCFH-DA assay-specific probes under a dark environment and upon illumination with blue light (405 nm) for 30 min. ESR spectra of DMPO-OOH adducts containing 1 M DMPO in aqueous solution with (b) different CNC-NCs before and after light irradiation and (c) CNC-AuNC@Ag at different light irradiation times including 1, 3, and 5 min. (d) ESR spectra of singlet oxygen adducts generated by CNC-AuNC@Ag in aqueous solutions containing 1 M TEMP before and after light irradiation.

The highest signal intensity was perceived for CNC-AuNC@Ag followed by CNC-AuNC@AgPd. Indeed, the highest efficacy can be attributed to the lifetime of the T1 state (Figure 3, Table 1), which decreases in the same order as the dopant element, e.g., Ag > AgPd > AgPt. In fact, longer residence in the triplet state increases the probability of energy transfer for ROS generation. The signal intensity was also observed to be directly proportional to the irradiation time, irrespective of the dopant elements (Figures 5c and S10a–c, Supporting Information). Analogous to DMPO, we have employed 2,2,6,6-tetramethyl-4-piperidone (TEMP) as a radical scavenger for the detection of singlet oxygen (^1^O_2_).^28^ However, no evident signal was observed from ^1^O_2_, declining the Type-II PD effect (Figures 5d and S10d–f). So, we can conclude that, because the O/R band has a strong triplet character and as its population dynamics is governed by ISC, the measured lifetime τ_R_ sets the time window for Type-I electron transfer to generate O_2_^–·^/^·^OH. Accordingly, the τ_R_ ordering (CNC-AuNC@Ag > CNC-AuNC@AgPd > CNC-AuNC@AgPt) reflects the ROS generation efficiency.

**Table 1:** Characteristic Timescales of the photocycles for CNC-NCs. For a Biexponential Process, the Reported Value Represents the Weighted Average of the Two Components.

**sample**	τ_2_’ (s)	τ_G_ (s)	τ_R_ (s)
CNC-AuNC	3.00 × 10^–12^	4.03 × 10^–9^	2.45 × 10^–6^
CNC-AuNC@Ag	2.53 × 10^–11^	3.02 × 10^–9^	2.52 × 10^–6^
CNC-AuNC@AgPd	5.33 × 10^–11^	6.35 × 10^–9^	1.21 × 10^–6^
CNC-AuNC@AgPt	8.10 × 10^–11^	6.08 × 10^–9^	1.04 × 10^–6^

### 2.4 Cytotoxicity, Biomedical Imaging, and PDT

Having clarified the structure and photophysical properties of CNC-NCs, we investigated their biomedical applications for theranostics. To ensure the cell permeability and imaging capability of these CNC-NC nanocomposites, A549 carcinoma epithelial cells were treated with their aqueous suspension (100 μM with respect to the Au atoms) and incubated for 24 h. The results demonstrate that the NCs were internalized by the cells into the cytoplasm and emitted bright luminescence under 458 nm excitation (Figure 6a,c,e). Corresponding z-stack images further confirm the significant cellular uptakes for all of the NCs (Figure 6b,d,f).

**Figure 6:**
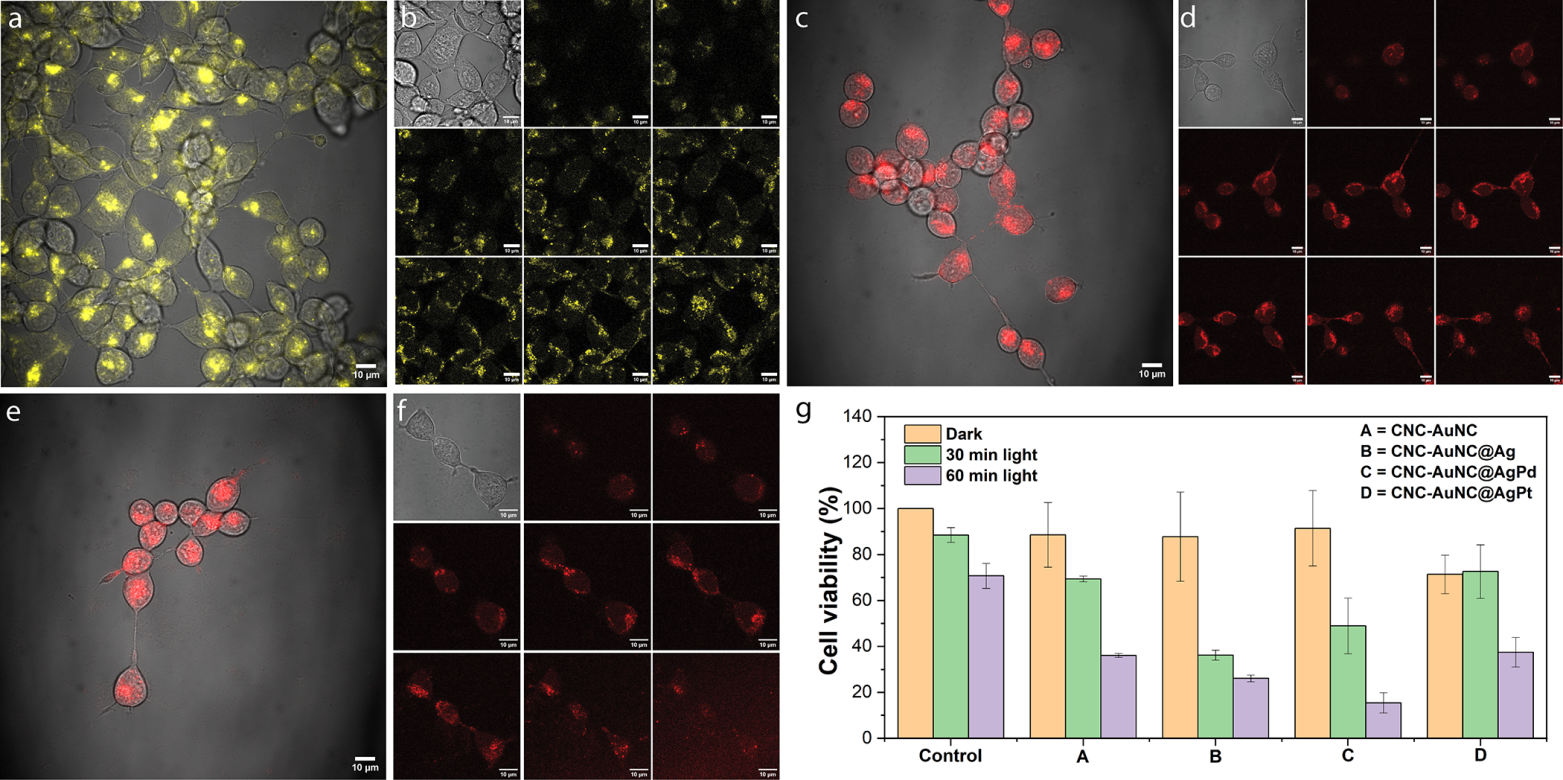
(a, c, e) Representative microscopy images (overlapped bright field and confocal) and (b, d, f) *z*-stacks (2.0 μm intervals) of A549 carcinoma epithelial cells incubated with CNC-NCs (100 μM) for 24 h, λ_ex_ = 458 nm. Incubated CNC-NCs are (a, b) CNC-AuNC@Ag, (c, d) CNC-AuNC@AgPd, and (e, f) CNC-AuNC@AgPt. (g) Photodynamic effect of the different types of CNC-NCs on murine sarcoma S180 cells under 350 nm excitation for 0, 30, and 60 min.

To address the cytotoxicity of CNC-NCs, cell counting kit 8 (CCK-8) assays were performed on NIH3T3 cells, in which no obvious cytotoxicity was observed under the experimental conditions (Figure S11, Supporting Information). To evaluate the PD effect of the CNC-NCs at the cellular level, phototoxicity was tested in the S180 sarcoma cell line under UV irradiation at 350 nm excitation (Figure 6g). For this experiment, the CCK-8 assay was performed with 250 μM CNC-NCs (with respect to the Au or metal atoms). All CNC-NCs, irrespective of the dopant elements (except CNC-AuNC@AgPt), show negligible toxicity to S180 cells in dark conditions. However, under light irradiation, the cell viability gradually decreased with an increase in the exposure time. After 30 min of continuous irradiation, CNC-AuNC@Ag shows the highest PD efficacy compared to the undoped CNC-AuNCs, followed by CNC-AuNC@AgPd. In contrast, the cell viability reaches a minimum within 60 min of irradiation using CNC-AuNC@AgPd as the PS. The results indicate that CNC-AuNC@Ag and CNC-AuNC@AgPd can kill cancer cells with a high efficacy. This is presumably due to the highest amount of ROS productions by the nanocomposites under the experimental conditions via PDT-I with the formation of O^2–·^ or ^·^OH radicals, favored by a higher lifetime of the triplet state where the photoinduced reaction takes place.

We further tested the effects of isolated AuNCs and CNC-AuNC@Ag composites on the 786-O cells (Figure 7a). To test cytotoxicity, 10,000 cells were seeded in each well of 12-well plates (Corning). Twenty-four h after seeding, the medium in selected wells was replaced with medium containing 100 μM of either AuNCs or CNC-NCs (relative to metal atom concentration). Afterward, the cell proliferation was monitored based on confluency using an Incucyte S3 Live-Cell Analysis System (Essen BioScience, Inc.). It was not possible to assay cell confluency in the case of CNC-NCs due to the high background signal from the CNC substrates. Therefore, we have used the 786-O PG cell line, which stably expresses the palmitoylated form of green fluorescent protein (GFP) and used the GFP count as an indicator of proliferation. While both AuNCs without light exposure and light exposure without AuNCs have little to no effect on the proliferation of 786-O PG cells, a strong reduction in proliferation has been observed under 405 nm LED light (310 mW) exposure in the presence of AuNCs (Figures 7a and S12, Supporting Information). Subsequently, the effect of CNC-AuNC@Ag on cell proliferation was tested (Figure 7b–e). The results demonstrate that the cells in the wells containing CNC-AuNC@Ag and exposed to blue light show the highest reduction in proliferation compared to both AuNCs and control. The results signify that while CNC-NC composites are highly biocompatible, they can generate ROS through the formation of O_2_^–·^ or ^·^OH radicals upon exposure to UV or blue light, enabling them to destroy the cells completely through the Type-I PD effect.

**Figure 7:**
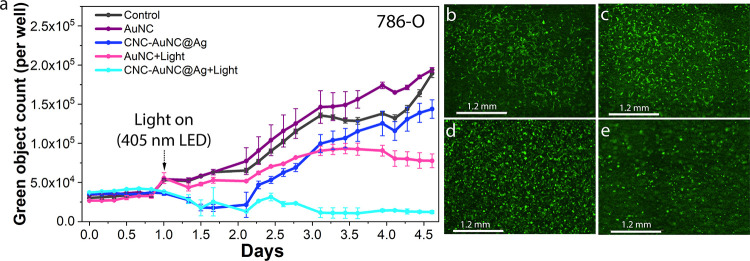
(a) Growth curves of 786-O PG cells treated with either AuNCs or CNC-NCs calculated based on the GFP count. (b–e) Representative green fluorescence images of 786-O PG cells before and after LED exposure. (b) Untreated 786-O PG cells before LED exposure. (c) Untreated 786-O PG cells after LED exposure. (d) CNC-AuNC@Ag-treated 786-O PG cells before LED exposure. (e) CNC-AuNC@Ag-treated 786-O PG cells after LED exposure.

After in vitro studies, we have tested the photodynamic effect on an ex ovo chicken embryo xenotransplantation model that has been developed in our lab.^46^ The main advantage of chicken embryo culture is that the cells, organoids, and pieces of tissues of different origins can be transplanted onto the CAM (chorioallantoic membrane), where they become vascularized without eliciting immune rejection.^47,48^ Xenotransplantation into chicken embryos is perfect for primary screening experiments that could be performed in a high throughput way without losing the high degree of complexity of in vivo systems. Because of high levels of autofluorescence in chicken embryos, we have used neuroblastoma SH-SY5Y cells for ex ovo experiments. SH-SY5Y cells were genetically modified to express both eGFP and Firefly luciferase, making it possible to detect not only fluorescent but also bioluminescent signals.^49^ While we observe a decrease in the luciferase signal in the majority of control (untreated) spheroids after 2 days ex ovo, spheroids treated with CNC-AuNCs@Ag and light (405 nm) completely lost bioluminescence at the same time point (Figure 8). To detect the mechanism of PDT-induced cell death, SH-SY5Y cells were stained with an Annexin V apoptosis marker. While control (untreated) cells as well as cells treated only with CNC-AuNCs@Ag or exposed only to light did not show an increase in the Annexin V red fluorescent signal, the cells treated both with CNC-AuNCs@Ag and light were characterized by high levels of apoptosis (Figure S13, Supporting Information).

**Figure 8:**
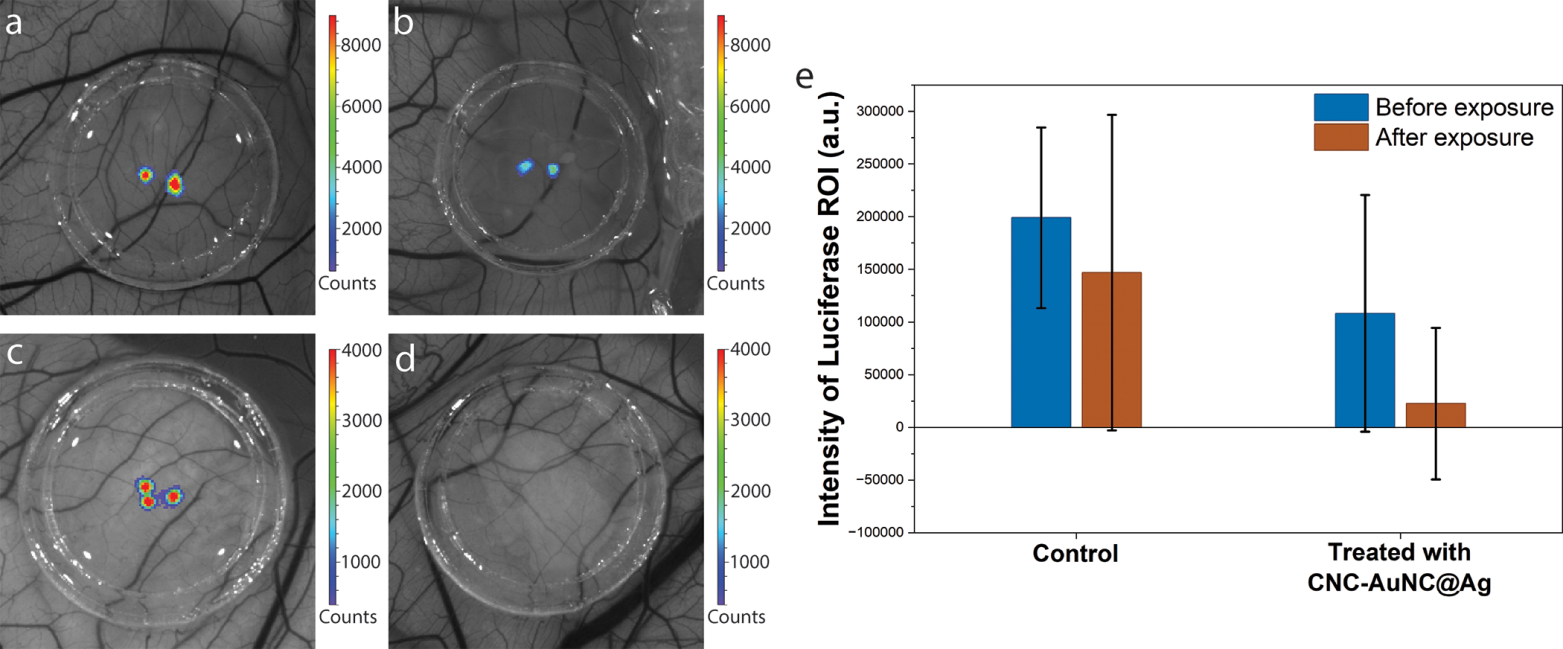
Representative bioluminescent images of SH-SY5Y spheroids placed on the CAM ex ovo: (a) control before exposure of light, (b) control after 2 days of light exposure, (c) treated with AuNCs@Ag, and (d) treated with AuNCs@Ag after 2 days of light exposure. (e) Quantification of bioluminescent signals from all the spheroids used. Bioluminescent signals were taken and quantified using the in vivo imaging system Caliper IVIS Spectrum.

## 3 Conclusions

In conclusion, we have developed a versatile nanoplatform for enhanced PDT by devising a synthesis method of gold-based alloy nanoclusters supported by bioderived cellulose nanocrystals, acting as efficient photosensitizers. These nanoclusters were modified with a trace amount of heteroatoms like Ag, Pd, and Pt to engineer their electronic level structures, photoluminescence properties, and excited-state relaxation pathways. The CNC-NC composites show high biocompatibility with intense fluorescence intensity after internalization into the cell cytoplasm, making them ideal candidates for theranostics. Although both AuNC and CNC-NC composites exhibit minimal cytotoxicity in the absence of light, CNC-AuNC@Ag and CNC-AuNC@AgPd show a significant reduction in cell proliferation under exposure to blue light, far greater than that observed with AuNCs alone·. This is probably due to the synergistic effect of NCs and CNCs in which the CNC matrix could absorb the light in the same region and transfer it to the NCs with minimal losses. As a result, the favorable photophysical properties of the NCs over the matrix are capable to generate effectual free radicals (O_2_^–·^ and ^·^OH) upon exposure to UV or visible light, leading to the destruction of cells through the Type-I photodynamic effect.

## 4 Experimental Section

### 4.1 Materials

HAuCl_4_·3H_2_O, PdCl_2_, H_2_PtCl_6_, glutathione, hydrochloric acid, sulfuric acid (64%), 5,5-dimethyl-1-pyrroline*n*-oxide (DMPO), and 2,2,6,6-tetramethyl-4-piperidone (TEMP) were purchased from Sigma-Aldrich and used as received. Whatman 1 and Whatman 541 filter papers and Spectra/Por 1 standard dialysis tubing (*M*_w_ cutoff 6–8 kDa) used in the CNC preparation were purchased from VWR. Ultrapure Milli-Q water (18 Ω) was used in all experiments.

### 4.2 Synthesis of CNCs

Sulfonate-ester functionalized CNCs were prepared through the acid hydrolysis of cotton filter paper (Whatman No. 1), following a previously reported procedure.^50^ Briefly, the filter paper was mechanically ground into a powder. Fifteen mg of this fine powder underwent hydrolysis with sulfuric acid (64%, 300 mL) at 45 °C for 45 min under gentle stirring (32 rpm). The reaction was halted by diluting the solution 10-fold with deionized water and allowing it to stand for 20 h. The supernatant was then discarded, and the residual CNC suspension underwent two washes with deionized water, followed by centrifugation. This suspension was further purified through dialysis in deionized water until the conductivity of the dialysate was less than 5 μS cm^–1^. Finally, the CNC suspension was filtered through a Whatman 541 filter paper and stored at 4 °C for subsequent use.

### 4.3 Synthesis of AuNCs

The synthesis of AuNCs was done by the procedure outlined by Luo et al.^51^ In a nutshell, 500 μL (20 × 10^–3^ M) of an aqueous solution of HAuCl_4_·3H_2_O and 150 μL (100 × 10^–3^ M) of glutathione in water were simultaneously introduced into 4.35 mL of Milli-Q (18 Ω) water at 25 °C, employing gentle stirring with a magnetic stirrer. Stirring was continued for an additional 15 min until a colorless solution was achieved. The reaction mixture was then subjected to heating at 70 °C for 24 h in an oil bath, with constant stirring at 500 rpm. Ultimately, the solution was cooled to room temperature and stored at 4 °C.

### 4.4 Synthesis of CNC-AuNCs and Metal-Doped CNC-AuNCs

The syntheses of CNC-AuNCs and metal-doped CNC-NCs were performed according to our previous procedure.^36^ Briefly, 500 μL (20 × 10^–3^ M) aqueous solution of HAuCl_4_·3H_2_O was mixed with 4.35 mL of an aqueous CNC dispersion (14.5 mg/mL) under gentle stirring. For the synthesis of CNC-AuNC@Ag, CNC-AuNC@Pd, and CNC-AuNC@Pt, 50 μL (20 × 10^–3^ M) of AgNO_3_, H_2_PdCl_4_, and H_2_PtCl_6_ were simultaneously introduced with 450 μL (20 × 10^–3^ M) of HAuCl_4_ into the aqueous CNC solution. Stirring persisted for 1 h to facilitate the absorption of Au (III) ions onto the negatively charged surface of CNCs. Subsequently, 150 μL (100 × 10^–3^ M) of glutathione aqueous solution was introduced to the reaction mixture, and stirring continued for an additional 15 min, followed by stirring at 70 °C for 24 h. After cooling the solution to room temperature, the product was isolated through centrifugation at 4500 rpm for 3 h. The supernatant was discarded, and the residue (CNC-NCs) was promptly mixed with 5 mL of water, followed by vortexing to achieve a colloidal dispersion. The resulting dispersion was stored at 4 °C for future use.

### 4.5 Optical Absorption

Steady-state absorption spectra were acquired at room temperature on diluted aqueous solutions of CNC-NCs by an AVANTES optical fiber spectrophotometer based on a multichannel CMOS detector.

### 4.6 Steady-State Photoluminescence

Steady-state photoluminescence spectra were recorded at room temperature from diluted aqueous solutions of CNC-NCs in a 1 cm cuvette by exciting each sample with 0.1 mJ, 5 ns laser pulses at 410 nm (obtained from a tunable laser) and dispersing their PL on an intensified CCD camera. The camera was triggered to acquire spectra within a window of 100 μs (sufficient to collect the whole emission) and slightly delayed (1 ns) with respect to the exciting pulse to exclude scattering effects. PLE spectra were measured by an Edinburgh Instruments FLS1000.

### 4.7 Nanosecond Time-Resolved Spectroscopy

Nanosecond-resolved PL measurements were performed via the same setup described in Section 4.6, by appropriately varying the width and delay of the measurement window in order to follow the PL decay. Kinetic traces were obtained by evaluating the integrated photoluminescence signal as a function of the delay time and fitted via a biexponential decay model in the form: *y*(*t*) = *A*_1_exp­(−*t*/τ_1_) + A_2_exp­(−*t*/τ_2_).

### 4.8 Transient Absorption Spectroscopy

TA (pump–probe) measurements were performed on the home-built setup described elsewhere,^52^ pumped via the 50 fs, 800 nm laser pulses (fwhm = 30 nm, 350 mJ energy per pulse) produced by a 5 kHz Ti:sapphire femtosecond amplifier (Spectra Physics Solstice-Ace). Said pulses were split 80%/20% via a beam splitter in order to generate the 400 nm pump and broadband probe, respectively. The former was obtained via second harmonic generation (20% efficiency, 50–100 nJ/pulse) in a 250 mm β-BBO crystal, subsequently isolated from the residual fundamental by a Schott BG40 filter, and chopped at 500 Hz, whereas the latter was obtained by focusing the 800 nm beam on a 1 mm quartz cell containing D_2_O, generating a “white light” pulse extending from 400 to 750 nm. Both the pump and probe were subsequently focused using a single parabolic mirror in such a way that they overlapped within the sample, which was continuously flowing in a 0.2 mm thick flow cell. The pump–probe delay was controlled by a motorized delay stage placed on the pump arm. The transmitted probe was then dispersed by a home-built monochromator (3 nm resolution) and focused on a 1024-pixel detector (Glaz Linescan-I) with single shot capabilities. In a typical measurement, the signal was obtained by averaging 5000 pumped and unpumped spectra for each pump–probe delay, with the delay scanned at least 10 times. The raw data were then subjected to correction procedures aimed at eliminating the effects of cross-phase modulation (XPM) and group velocity dispersion (GVD), thereby obtaining the final data presented in the paper, which have a temporal resolution of about 80 fs. The decay kinetics, extracted analogously to the nanosecond-resolved ones, were fitted by a multiexponential decay model convoluted with a (Gaussian) instrumental response function.

### 4.9 Absolute Quantum Yield Measurements

Absolute QY measurements were performed according to the protocol reported elsewhere.^53^ The aqueous solutions of CNC-NCs were placed in thin silica tubes, inserted into a Labsphere integrating sphere, and excited (both directly and indirectly) via a 405 nm CW laser diode. The integrating sphere signal was then collected via the AVANTES multichannel spectrometer already described in Section 2.1.

### 4.10 Atomic Absorption Spectrometry (AAS)

The samples were diluted with 2% HNO_3_ to dilution ratios of 1:10 (Ag, Pt, and Pd) and 1:100 (Au). The elements were analyzed from the diluted solution samples with AAS using ThermoScientific ICE 3000.

### 4.11 Inductively Coupled Plasma Mass Spectrometry (ICP-MS)

ICP-MS was performed by a ThermoScientific ICP-SFMS ELEMENT XR inductively coupled plasma mass spectrometer. This analysis was performed in accordance with the standards SS-EN ISO 17294–2:2023 and US EPA Method 200.8:1994 in a laboratory accredited to ISO 17025. The testing was conducted by the laboratory service provider, Measurlabs.

### 4.12 DLS and Zeta Potential Measurements

The measurements were performed by a Zetasizer Nano ZS 90 (Malvern Instruments, UK) at room temperature. The instrument was equipped with a 633 nm red laser and 90° detection optics, which measure the particle size in the range from 0.3 nm to 5 μm. Samples were adjusted to different pH values using 0.1 M HCl and 0.1 M NaOH solutions.

### 4.13 X-ray Photoelectron Spectroscopy

The measurements were conducted using a Kratos AXIS Ultra DLD X-ray photoelectron spectrometer, employing a monochromated AlKα X-ray source with an energy of 1486.7 eV, operated at 100 W. For the survey spectra, a pass energy of 80 eV and a step size of 1.0 eV were utilized, while the high-resolution spectra employed a pass energy of 20 eV and a step size of 0.1 eV. Photoelectrons were gathered at a 90° takeoff angle within ultrahigh vacuum conditions, maintaining a base pressure typically below 1 × 10^–9^ Torr. The X-ray beam spot diameter was 1 mm, and the analysis area for these measurements was set at 300 μm × 700 μm. Multiple spots on each sample surface were probed for both survey and high-resolution spectra to ensure homogeneity and to identify any surface charge effects. The high-resolution spectra of the samples endured charge correction with respect to the position of the C–O bonding of carbon, set at 286.5 eV.

### 4.14 Scanning Transmission Electron Microscopy (STEM)

STEM of CNC-NCs was conducted using a JEM-2200FS Double Cs-corrected transmission electron microscope operating at an acceleration voltage of 200 kV with field-emission guns. For STEM analyses, specimens were prepared by drop-casting from aqueous dispersions onto ultrathin-carbon-coated copper grids with a thickness of less than 10 nm, followed by a 1 min incubation period, after which excess material was removed using a filter paper. The dimensions of the NCs over CNC surfaces were determined from the micrographs by using ImageJ software.

### 4.15 Electron Spin Resonance (ESR) Spectroscopy

To detect free radicals by electron spin resonance (Magnettech, Bruker, MS5000X V5.7), 40 μL of 1.0 M 5,5-dimethyl-1-pyrroline n-oxide (DMPO) in water was added to 500 μL of 250 μM of different CNC-NCs. The mixture was then irradiated with 405 nm light and measured using an ESR spectrometer. To detect singlet oxygen, 40 μL of 1.0 M 2,2,6,6-tetramethyl-4-piperidone (TEMP) was added to the same amount of different CNC-NCs and then exposed to 405 nm light during the measurement. Then, the experiment was repeated under different light irradiation times (1, 3, and 5 min).

### 4.16 Computational Methods

All of the calculations were done using a DFT code GPAW.^54^ The wave functions were described with real-space uniform grids. The grid spacing was 0.2 Å in this work. PBE functional was used for the exchange-correlation energy^55^ and the van der Waals interactions were described by the Tkatchenko–Scheffler model.^56^ Per atom, the electronic configuration of valence electrons is Au­(5d^10^6s^1^), Ag­(4p^6^4d^10^5s^1^), Pt­(5p^6^5d^9^6s^1^), Pd­(4p^6^4d^10^), C­(2s^2^2p^2^), and H­(1s^1^). The remaining electrons were treated as a frozen core. The geometry was considered to be converged during the structure optimization when the maximum residual force was below 0.05 eV/Å.

### 4.17 Cell Imaging

Human epithelial carcinoma cells (A549) were used to assess the internalization of CNC-NCs (a generous gift from Prof. Kostiainen, Aalto University). The cells were seeded in an observation chamber (μ-slide Angiogenesis, Ibidi) containing Dulbecco’s modified Eagle medium (DMEM) supplemented with 10% fetal bovine serum (FBS) and antibiotics (100 μg/mL streptomycin and 100 U/mL penicillin). After 1 day in an incubator (ICO50 CO_2_ incubator, Memmert Co.) at 37 °C under a 95% air/CO_2_ atmosphere, the cell culture medium was removed. The cells were washed once with phosphate-buffered saline (PBS) and incubated with the desired CNC-NC solution (100 μM with respect to the Au or metal atoms). After an additional day in the incubator, the solution was discarded, and the cells were washed once with PBS. Finally, the well was completely filled with PBS, covered by a glass slide, and the cells were imaged upside down.

Cell imaging was performed using a confocal microscope (LSM 710, 60×/1.4 oil immersion objective, λ_ex_ = 458 nm, λ_em_ = 463–735 nm). Images were exported from the instrument software (Zeiss Zen Black) in the CZI format and further processed with Fiji.

### 4.18 Cytotoxicity Studies

#### 4.18.1 Without UV Exposure

A cell counting kit (CCK-8, Sigma-Aldrich Co.) was used to evaluate the effect of gold clusters on the viability of murine sarcoma cells (S180) and noncancerous murine fibroblast cells (NIH3T3). The kit was used according to the manufacturer’s instructions. A cell suspension (100 μL, 5000 cells per well) was dispersed in a 96-well plate containing DMEM medium supplemented with 10% FBS and antibiotics (100 μg mL^–1^ streptomycin and 100 U mL^–1^ penicillin). The plate was preincubated at 37 °C under a 95% air/5% CO_2_ atmosphere for 1 day. The medium was then changed with a solution containing cell medium, PBS, distilled water, and the desired gold cluster solution to achieve Au concentrations *C* of 0, 10, 50, 100, 150, 200, 250, 500, 750, and 1000 μM. The plate was then incubated for 1 day under the same conditions as above. The solution in each well was replaced with 100 μL of supplemented DMEM, and the absorbance at 450 nm of each well was measured at *t* = 0 (*t*_0_) using a Synergy H1 hybrid microplate reader (BioTek). CCK-8 solution (10 μL) was then added to each well, and the plate was put in the incubator for 2 to 4 h. The absorbance at 450 nm was read again (*t*_1_). Each sample was tested at least three times.

$$\mathrm{Cell}\;\mathrm{viability},C(\%)=100\times \frac{A_{C,t_{1}}-A_{C,t_{0}}}{A_{C=0,t_{1}}-A_{C=0,t_{0}}}$$

#### 4.18.2 With UV Exposure

CCK-8 was used to evaluate the effect of Au plus UV light on the viability of S180 and NIH3T3 cell lines. The kit was used according to the instructions of the manufacturer. A cell suspension (200 μL, 10,000 cells per well) was dispersed in a 48-well plate containing the supplemented DMEM medium. The plate was preincubated in the incubator for 1 day. The medium was then replaced with a solution containing cell medium, PBS, distilled water, and the desired CNC-NC solution to achieve the Au concentration C of 250 μM. The plate was then incubated for 1 day under the same conditions as described above. The solution in each well was replaced with 200 μL of supplemented DMEM and half of the well plate was then covered with aluminum foil to protect it from UV. The well plate was then exposed to UV light (λ_ex_ = 350 nm, distance from UV source = 25 cm, power = 12 W) for 30 or 60 min. Then, the absorbance of each well at 450 nm was measured at *t* = 0 (*t*_0_) using the microplate reader. CCK-8 solution (20 μL) was then added to each well, and the plate was incubated between 2 and 4 h in the incubator. The absorbance at 450 nm was then read again (*t*_1_). Each sample was tested at least three times.

$$\mathrm{Cell}\;\mathrm{viability},C(\%)=100\times \frac{A_{C,t_{1}}(\mathrm{UV})-A_{C,t_{0}}(\mathrm{UV})}{A_{C=0,t_{1}}(\mathrm{no}\;\mathrm{UV})-A_{C=0,t_{0}}(\mathrm{no}\;\mathrm{UV})}$$

### 4.19 Photodynamic Therapy on Human Renal Cell Carcinoma

The human 786-O cells with stable expression of palmitoylated green fluorescent protein (PG) were cultured at 37 °C in RPMI 1640 medium (Biowest) supplemented with 10% fetal bovine serum (Gibco, Grand Island, NY, USA) and 1% Penicillin/Streptomycin (Thermo Fisher Scientific, USA).

We tested the effect of CNC-NCs under blue-light exposure (M405L4-C2–405 nm, 310 mW Collimated LED for Leica DMI, 1000 mA) on the proliferation of 786-O PG. 10,000 cells were seeded into each well of the 12-well cell culture plate (Corning). Twenty-four h post seeding, the media in selected wells were replaced with media containing 100 μM of different CNC-NCs (with respect to the Au or metal atoms). The cells were further incubated for 24 h, after which the wells were exposed to light for 30 min. The proliferations of 786-O PG cells were assayed for several days after light exposure by measuring the green fluorescence using interval imaging analysis by an Incucyte S3 Live-Cell Analysis System (Essen BioScience, Inc.).

### 4.20 Detection of Intracellular Reactive Oxygen Species (ROS)

ROS production was evaluated in 786–0 cells by using a DCFH-DA assay-specific probe kit (Abcam, ab113851). The cells were seeded in DMEM medium supplemented with 10% fetal bovine serum (FBS) and antibiotics (100 μg/mL streptomycin and 100 U/mL penicillin) at a density of 30,000 cells per well in a 96-well plate (Corning, 3599) and incubated overnight at 37 °C in a CO_2_ incubator. The following day, the culture medium was replaced with fresh medium, and nanoclusters or CNC-NCs were added to each well to achieve a final concentration of 100 μM. After 24 h of incubation, the cells were washed once with phosphate-buffered saline (PBS). After that, 100 μL of 25 μM 2’,7’-dichlorofluorescin diacetate (DCFH-DA) solution was added to each well, and the plate was incubated at 37 °C for 45 min in the dark. The cells were washed once with a supplemented buffer, after which 100 μL of this buffer containing 10% fetal bovine serum was added to each well. The cells were then exposed to blue light for 30 min. Cell imaging and quantification of the total green fluorescence integrated intensity were performed using the IncuCyte S3 system.

To detect ROS production with confocal microscopy, 786–0 cells were seeded at a density of 30,000 cells per well in 8-well IBIDI 80826 plates. The following day, the culture medium was changed, and CNC-AuNC@Ag was added to each well with a final concentration of 100 μM. After 24 h incubation, the cells were washed with PBS followed by the addition of 100 μL of DCFH-DA solution (25 μM). After incubation at 37 °C for 45 min in the dark, the cells were washed again with 1× buffer solution. Subsequently, 100 μL of the supplemented buffer containing 10% fetal bovine serum was added to each well. The cells were then exposed to blue light (405 nm) for 30 min. Confocal fluorescence images were taken by a Leica SP8 FALCON confocal microscope using a 20 × water immersion objective with excitation and emission wavelengths of 485 and 535 nm, respectively.

### 4.21 Culturing Neuroblastoma SH-Sy5y Cells

Neuroblastoma cells SH-SY5Y, genetically modified to express eGFP and Firefly luciferase,^49^ were cultured in a 1:1 mixture of MEM (Merck, M4655–500 ML) and F-12 Nutrient Mix (Gibco, 11765054) supplemented with 10% FBS and 1% penicillin/streptomycin/glutamine (Gibco, 10378016) in a 5% CO_2_ incubator at 37 °C. Cell numbers and viability were tested with Trypan Blue Stain (T10282 Invitrogen) in a TC20 Automated Cell Counter (145–0101 Biorad); viability was in the range of 95–97%.

### 4.22 Apoptosis Detection

Apoptotic cells were detected by using Annexin V (Sartorius, 4641) red dye staining. Neuroblastoma cells were plated into glass bottom ibidi μ-Slide 8-well plates (in 200 μL of MEM/F-12) and cultured for 2 days. After that, cells were treated with CNC-AuNCs@Ag solution (1:10 dilution), and the next day, they were exposed to light for 30 min. Annexin V was added to the cells immediately after light exposure in a 1:400 dilution. Cells were imaged using a Zeiss LSM 780 confocal microscope 30 min after staining with Annexin V and on day 3.

### 4.23 Chicken Embryo Ex Ovo Culture

The fertilized Hy-line White (Hy-line International) eggs were acquired from Haaviston Siitoskanala, Panelia, Finland. Upon arrival, the fertilized eggs were kept in a refrigerator at 15 °C. Three days prior to starting ex ovo cultures, the eggs were placed in a humified incubator at 37.5 °C on an automated egg turner. On day 3 of embryonic development, the eggs were carefully opened by making a vertical crack in the shell on the dorsal side of the embryo.^46^ The contents of the eggs were carefully transferred to a glass bowl filled with water and covered with a plastic cling to create a hammock-like waterbed. The glass bowl was then covered with a Petri dish to reduce water loss through evaporation. After that, ex ovo cultures were kept in a 5% CO_2_ incubator at 37 °C.

### 4.24 Preparation of Neuroblastoma Spheroids

For preparation of SH-SY5Y spheroids, 5 × 10^4^ cells were seeded in 200 μL of medium per well in a 96-well ultralow adherent plate (ThermoScientific, 174925) and centrifuged for 3 min at 1000 rpm (M10 Microplate Swinging Bucket Rotor for ThermoScientific SL8 centrifuge). Spheroids were kept for 2–3 days in a 5% CO_2_ incubator at 37 °C before transfer to the CAM.

### 4.25 Xenotransplantation of SH-SY5Y Spheroids on the CAM and PDT Experiments

The membrane rings used in the CAM transplantation were prepared from 12-well cell culture inserts with a 0.4-μm-pore poly­(ethylene terephthalate) (PET) membrane. The sides of the cell culture inserts were trimmed to a height of 2–3 mm using a handsaw. The insets were sterilized using 70% ethanol. The membrane reservoir was then rinsed with PBS and the culture medium. The spheroids were then gently placed onto the membrane in 200 μL medium and allowed to attach in a CO_2_ incubator for 1–2 h. After that, the spheroid-containing membrane rings were carefully placed onto the CAM (on days 7–8 of embryonic development). The next day, spheroids were treated with 20 μL of CNC-AuNCs@Ag solution, and the next day, they were exposed to light for 30 min. Spheroids were observed under an Olympus SZ61 fluorescent stereomicroscope to detect the GFP signal (Supplementary Videos S1 and S2) and under an IVIS spectrum imaging chamber to detect the luciferase signal (Figure 8).

### 4.26 Bioluminescence Detection Using the IVIS Spectrum

Bioluminescence imaging was used to quantify the size of the tumor spheroids before and 2 days after light treatment. Luciferin (10 μL of 30 mg/mL solution per ring) was added to membrane rings containing spheroids approximately 30 min prior to imaging. The bioluminescence signal was detected and quantified using the in vivo imaging system Caliper IVIS Spectrum. During the procedure, embryos were carefully placed inside the IVIS Spectrum imaging chamber. Parameters such as focus, binning, exposure time, and field of view were optimized to ensure high-quality image acquisition.

## Supplementary Material







## Notes

General view of chicken embryo ex ovo culture 4 days after transplantation of membrane rings with SH-SY5Y neuroblastoma spheroids (MOV)
General view of chicken embryo ex ovo culture 4 days after transplantation of membrane rings with SH-SY5Y neuroblastoma spheroids and 3 days after addition of CNC-AuNC@Ag (MOV)
AAS and ICP-MS analysis, DLS and zeta potential measurements, XPS spectra, UV–vis spectra, PLE spectra, DFT studies, decimal semilogarithmic plots, fitted TA kinetic traces, ESR spectra, cell viabilities, confocal microscopy images for ROS production, the growth curves of 786-O PG cells and apoptosis detection (PDF)

## References

[1] Lucky S. S., Soo K. C., Zhang Y. (2015). Nanoparticles in Photodynamic Therapy. Chem. Rev..

[2] Wang R., Li X., Yoon J. (2021). Organelle-Targeted
Photosensitizers for Precision Photodynamic
Therapy. ACS Appl. Mater. Interfaces.

[3] Celli J. P., Spring B. Q., Rizvi I., Evans C. L., Samkoe K. S., Verma S., Pogue B. W., Hasan T. (2010). Imaging and Photodynamic
Therapy: Mechanisms, Monitoring, and Optimization. Chem. Rev..

[4] Pham T. C., Nguyen V. N., Choi Y., Lee S., Yoon J. (2021). Recent Strategies
to Develop Innovative Photosensitizers for Enhanced Photodynamic Therapy. Chem. Rev..

[5] Zhou Z., Song J., Nie L., Chen X. (2016). Reactive Oxygen
Species
Generating Systems Meeting Challenges of Photodynamic Cancer Therapy. Chem. Soc. Rev..

[6] Liu P., Yang W., Shi L., Zhang H., Xu Y., Wang P., Zhang G., Chen W. R., Zhang B., Wang X. (2019). Concurrent Photothermal
Therapy and Photodynamic Therapy for Cutaneous
Squamous Cell Carcinoma by Gold Nanoclusters under a Single NIR Laser
Irradiation. J. Mater. Chem. B.

[7] Xie J., Wang Y., Choi W., Jangili P., Ge Y., Xu Y., Kang J., Liu L., Zhang B., Xie Z., He J., Xie N., Nie G., Zhang H., Kim J. S. (2021). Overcoming
Barriers in Photodynamic Therapy Harnessing Nano-Formulation Strategies. Chem. Soc. Rev..

[8] Chen J., Fan T., Xie Z., Zeng Q., Xue P., Zheng T., Chen Y., Luo X., Zhang H. (2020). Advances in Nanomaterials
for Photodynamic Therapy Applications: Status and Challenges. Biomaterials.

[9] Hak A., Ali M. S., Sankaranarayanan S. A., Shinde V. R., Rengan A. K. (2023). Chlorin
E6: A Promising Photosensitizer in Photo-Based Cancer Nanomedicine. ACS Applied Bio Materials.

[10] Li X., Lee S., Yoon J. (2018). Supramolecular
Photosensitizers Rejuvenate Photodynamic
Therapy. Chem. Soc. Rev..

[11] Zhang X. Q., Yin L. H., Tang M., Pu Y. P. (2011). ZnO, TiO 2, SiO
2, and Al 2O 3 Nanoparticles-Induced Toxic Effects on Human Fetal
Lung Fibroblasts. Biomed. Environ. Sci..

[12] Andersson-Willman B., Gehrmann U., Cansu Z., Buerki-Thurnherr T., Krug H. F., Gabrielsson S., Scheynius A. (2012). Effects of
Subtoxic Concentrations of TiO2 and ZnO Nanoparticles on Human Lymphocytes,
Dendritic Cells and Exosome Production. Toxicol.
Appl. Pharmacol..

[13] Chandra S., Masuda Y., Shirahata N., Winnik F. M. (2017). Transition-Metal-Doped
NIR-Emitting Silicon Nanocrystals. Angewandte
Chemie - International Edition.

[14] Zhang X. D., Wu D., Shen X., Liu P. X., Fan F. Y., Fan S. J. (2012). In Vivo
Renal Clearance, Biodistribution, Toxicity of Gold Nanoclusters. Biomaterials.

[15] Zhu G. H., Gray A. B. C., Patra H. K. (2022). Nanomedicine:
Controlling Nanoparticle
Clearance for Translational Success. Trends
Pharmacol. Sci..

[16] Soo
Choi H., Liu W., Misra P., Tanaka E., Zimmer J. P., Itty Ipe B., Bawendi M. G., Frangioni J. V. (2007). Renal Clearance
of Quantum Dots. Nat. Biotechnol..

[17] van
de Looij S. M., Hebels E. R., Viola M., Hembury M., Oliveira S., Vermonden T. (2022). Gold Nanoclusters: Imaging, Therapy,
and Theranostic Roles in Biomedical Applications. Bioconjug. Chem..

[18] Cifuentes-Rius A., Deepagan V. G., Xie J., Voelcker N. H. (2021). Bright Future of
Gold Nanoclusters in Theranostics. ACS Appl.
Mater. Interfaces.

[19] Chandra S., Nonappa, Beaune G., Som A., Zhou S., Lahtinen J., Jiang H., Timonen J. V. I., Ikkala O., Ras R. H. A. (2019). Highly Luminescent Gold Nanocluster
Frameworks. Adv. Opt Mater..

[20] Song X., Zhu W., Ge X., Li R., Li S., Chen X., Song J., Xie J., Chen X., Yang H. (2021). A New Class
of NIR-II Gold Nanocluster-Based Protein Biolabels for In Vivo Tumor-Targeted
Imaging. Angewandte Chemie - International Edition.

[21] Dutta D., Sailapu S. K., Simon A. T., Ghosh S. S., Chattopadhyay A. (2019). Gold-Nanocluster-Embedded
Mucin Nanoparticles for Photodynamic Therapy and Bioimaging. Langmuir.

[22] Zhu H., Wang S., Wang Y., Song C., Yao Q., Yuan X., Xie J. (2022). Gold Nanocluster
with AIE: A Novel
Photodynamic Antibacterial and Deodorant Molecule. Biomaterials.

[23] Geng T., Zhao L., Wu D., Zhang H., Zhao X., Jiao M., Zeng L. (2021). Bovine Serum
Albumin-Encapsulated
Ultrasmall Gold Nanoclusters for Photodynamic Therapy of Tumors. ACS Appl. Nano Mater..

[24] Zhou S., Gustavsson L., Beaune G., Chandra S., Niskanen J., Ruokolainen J., Timonen J. V. I., Ikkala O., Peng B., Ras R. H. A. (2023). PH-Responsive
Near-Infrared Emitting Gold Nanoclusters. Angewandte
Chemie - International Edition.

[25] Huang P., Lin J., Wang S., Zhou Z., Li Z., Wang Z., Zhang C., Yue X., Niu G., Yang M., Cui D., Chen X. (2013). Photosensitizer-Conjugated
Silica-Coated Gold Nanoclusters
for Fluorescence Imaging-Guided Photodynamic Therapy. Biomaterials.

[26] Zhang C., Li C., Liu Y., Zhang J., Bao C., Liang S., Wang Q., Yang Y., Fu H., Wang K., Cui D. (2015). Gold Nanoclusters-Based
Nanoprobes for Simultaneous Fluorescence
Imaging and Targeted Photodynamic Therapy with Superior Penetration
and Retention Behavior in Tumors. Adv. Funct
Mater..

[27] Vankayala R., Kuo C. L., Nuthalapati K., Chiang C. S., Hwang K. C. (2015). Nucleus-Targeting
Gold Nanoclusters for Simultaneous in Vivo Fluorescence Imaging, Gene
Delivery, and NIR-Light Activated Photodynamic Therapy. Adv. Funct Mater..

[28] Han R., Zhao M., Wang Z., Liu H., Zhu S., Huang L., Wang Y., Wang L., Hong Y., Sha Y., Jiang Y. (2020). Super-Efficient in
Vivo Two-Photon Photodynamic Therapy
with a Gold Nanocluster as a Type i Photosensitizer. ACS Nano.

[29] Ho-Wu R., Yau S. H., Goodson T. (2017). Efficient
Singlet Oxygen Generation
in Metal Nanoclusters for Two-Photon Photodynamic Therapy Applications. J. Phys. Chem. B.

[30] Kim M. M., Ghogare A. A., Greer A., Zhu T. C. (2017). On the in Vivo Photochemical
Rate Parameters for PDT Reactive Oxygen Species Modeling. Phys. Med. Biol..

[31] Kang X., Li Y., Zhu M., Jin R. (2020). Atomically
Precise Alloy Nanoclusters:
Syntheses, Structures, and Properties. Chem.
Soc. Rev..

[32] Wang S., Li Q., Kang X., Zhu M. (2018). Customizing the Structure, Composition,
and Properties of Alloy Nanoclusters by Metal Exchange. Acc. Chem. Res..

[33] Jin R., Nobusada K. (2014). Doping and Alloying
in Atomically Precise Gold Nanoparticles. Nano
Res..

[34] Hossain S., Niihori Y., Nair L. V., Kumar B., Kurashige W., Negishi Y. (2018). Alloy Clusters: Precise Synthesis
and Mixing Effects. Acc. Chem. Res..

[35] Oh E., Delehanty J. B., Field L. D., Mäkinen A. J., Goswami R., Huston A. L., Medintz I. L. (2016). Synthesis and Characterization
of PEGylated Luminescent Gold Nanoclusters Doped with Silver and Other
Metals. Chem. Mater..

[36] Chandra S., Sciortino A., Shandilya S., Fang L., Chen X., Nonappa, Jiang H., Johansson L. S., Cannas M., Ruokolainen J., Ras R. H. A., Messina F., Peng B., Ikkala O. (2023). Core-Selective
Silver-Doping of Gold Nanoclusters by Surface-Bound Sulphates on Colloidal
Templates: From Synthetic Mechanism to Relaxation Dynamics. Adv. Opt Mater..

[37] Hynninen V., Chandra S., Das S., Amini M., Dai Y., Lepikko S., Mohammadi P., Hietala S., Ras R. H. A., Sun Z., Ikkala O., Nonappa (2021). Luminescent Gold Nanocluster-Methylcellulose
Composite Optical Fibers with Low Attenuation Coefficient and High
Photostability. Small.

[38] Kang X., Silalai C., Lv Y., Sun G., Chen S., Yu H., Xu F., Zhu M. (2017). Au15Ag3­(SPhMe2)­14 Nanoclusters –
Crystal Structure and Insights into Ligand-Induced Variation. Eur. J. Inorg. Chem..

[39] Xiang J., Li P., Song Y., Liu X., Chong H., Jin S., Pei Y., Yuan X., Zhu M. (2015). X-Ray Crystal Structure, and Optical
and Electrochemical Properties of the Au15Ag3­(SC6H11)­14 Nanocluster
with a Core-Shell Structure. Nanoscale.

[40] Kwak K., Tang Q., Kim M., Jiang D. E., Lee D. (2015). Interconversion
between Superatomic 6-Electron and 8-Electron Configurations of M@Au24­(SR)­18
Clusters (M = Pd, Pt). J. Am. Chem. Soc..

[41] Zhou M., Zhong J., Wang S., Guo Q., Zhu M., Pei Y., Xia A. (2015). Ultrafast Relaxation Dynamics of Luminescent Rod-Shaped,
Silver-Doped AgxAu25-x Clusters. J. Phys. Chem.
C.

[42] Alkan F., Pandeya P., Aikens C. M. (2019). Understanding the
Effect of Doping
on Energetics and Electronic Structure for Au25, Ag25, and Au38 Clusters. J. Phys. Chem. C.

[43] Liu Z., Luo L., Jin R. (2024). Visible to
NIR-II Photoluminescence of Atomically Precise
Gold Nanoclusters. Adv. Mater..

[44] Chandra S., Sciortino A., Das S., Ahmed F., Jana A., Roy J., Li D., Liljeström V., Jiang H., Johansson L. S., Chen X., Nonappa, Cannas M., Pradeep T., Peng B., Ras R. H. A., Sun Z., Ikkala O., Messina F. (2023). Gold Au­(I)­6
Clusters with Ligand-Derived Atomic Steric Locking: Multifunctional
Optoelectrical Properties and Quantum Coherence. Adv. Opt Mater..

[45] Ruckebusch C., Sliwa M., Pernot P., de Juan A., Tauler R. (2012). Comprehensive
Data Analysis of Femtosecond Transient Absorption Spectra: A Review. J. Photochem. Photobiol. C: Photochem. Rev..

[46] Kaisto S., Saarela U., Dönges L., Raykhel I., Skovorodkin I., Vainio S. J. (2020). Optimization of
Renal Organoid and Organotypic Culture
for Vascularization, Extended Development, and Improved Microscopy
Imaging. J. Vis. Exp..

[47] Kain K. H., Miller J. W. I., Jones-Paris C. R., Thomason R. T., Lewis J. D., Bader D. M., Barnett J. V., Zijlstra A. (2014). The Chick Embryo as
an Expanding Experimental Model for Cancer and Cardiovascular Research. Dev. Dyn..

[48] Villanueva, H. ; Sikora, A. G. The Chicken Embryo Chorioallantoic Membrane (CAM): A Versatile Tool for the Study of Patient-Derived Xenografts. In Mammary Stem Cells: Methods and Protocols; Vivanco, M. d. M. , Ed.; Springer US: New York, NY, 2022; 209–220. 10.1007/978-1-0716-2193-6_11 35175599

[49] Jahan F., Penna L., Luostarinen A., Veltman L., Hongisto H., Lähteenmäki K., Müller S., Ylä-Herttuala S., Korhonen M., Vettenranta K., Laitinen A., Salmenniemi U., Kerkelä E. (2024). Automated
and Closed Clinical-Grade Manufacturing Protocol Produces Potent NK
Cells against Neuroblastoma Cells and AML Blasts. Sci. Rep.

[50] Hynninen V., Hietala S., McKee J. R., Murtomäki L., Rojas O. J., Ikkala O., Nonappa (2018). Inverse Thermoreversible Mechanical
Stiffening and Birefringence in a Methylcellulose/Cellulose Nanocrystal
Hydrogel. Biomacromolecules.

[51] Luo Z., Yuan X., Yu Y., Zhang Q., Leong D. T., Lee J. Y., Xie J. (2012). From Aggregation-Induced
Emission
of Au­(I)–Thiolate Complexes to Ultrabright Au(0)@Au­(I)–Thiolate
Core–Shell Nanoclusters. J. Am. Chem.
Soc..

[52] Sciortino A., Ferrante F., Mauro N., Buscarino G., Sciortino L., Giammona G., Cannas M., Duca D., Messina F. (2021). Disclosing
the Emissive Surface Traps in Green-Emitting
Carbon Nanodots. Carbon N Y.

[53] De
Mello J. C., Wittmann H. F., Friend R. H. (1997). An Improved Experimental
Determination of External Photoluminescence Quantum Efficiency. Adv. Mater..

[54] Enkovaara J., Rostgaard C., Mortensen J. J., Chen J., Dułak M., Ferrighi L., Gavnholt J., Glinsvad C., Haikola V., Hansen H. A., Kristoffersen H. H., Kuisma M., Larsen A. H., Lehtovaara L., Ljungberg M., Lopez-Acevedo O., Moses P. G., Ojanen J., Olsen T., Petzold V., Romero N. A., Stausholm-Mo̷ller J., Strange M., Tritsaris G. A., Vanin M., Walter M., Hammer B., Häkkinen H., Madsen G. K. H., Nieminen R. M., No̷rskov J. K., Puska M., Rantala T. T., Schio̷tz J., Thygesen K. S., Jacobsen K. W. (2010). Electronic Structure Calculations
with GPAW: A Real-Space Implementation of the Projector Augmented-Wave
Method. J. Phys.: Condens. Matter.

[55] Perdew J. P., Burke K., Ernzerhof M. (1996). Generalized
Gradient Approximation
Made Simple. Phys. Rev. Lett..

[56] Tkatchenko A., Scheffler M. (2009). Accurate Molecular van Der Waals Interactions from
Ground-State Electron Density and Free-Atom Reference Data. Phys. Rev. Lett..

