# Supplementary material for: Synergistic Effects in Matrix-Embedded Alloy Nanoclusters: Advanced Type‑I Photosensitizers for Theranostics
Source: ACS Appl Mater Interfaces. 2026 Jan 28;18(5):8685–98. doi: 10.1021/acsami.5c22942 (PMC12903107; doi:10.1021/acsami.5c22942)
Supplement: Supplementary file 3 [file am5c22942_si_003.pdf]

Supporting Information

Synergistic effects in matrix-embedded alloy nanoclusters: Advanced Type-I photosensitizers for theranostics

Negar Hosseiniyan, [a] *Pietro Castronovo,* [b],[c] *Gregory Beaune,* [a] *Eslam Abdelrady,* [d] *Xi Chen,* [a] *Artem Zhyvolozhnyi,* [d] *Hamza Siddiqui,* [d] *Jahan Farhana,* [e] *Hua Jiang,* [a] *Minna Makki,* [a] *Marco Cannas,* [b],[c] *Alice Sciortino,* [b],[c] *Ilya Skovorodkin,* [d] *Anatoliy Samoylenko,* [d] *Seppo J. Vainio,* [d] *Fabrizio Messina* *[b],[c] *Sourov Chandra,**[a]

[a] Department of Applied Physics, Aalto University, P. O. Box 15100, FI-00076, Espoo, Finland.

[b] Dipartimento di Fisica e Chimica - Emilio Segrè, Università degli Studi di Palermo, Via Archirafi 36, 90123 Palermo, Italy.

[c] ATeN Center, Università degli studi di Palermo, viale delle scienze, Edificio 18, 90128, Palermo, Italy

[d] Laboratory of Developmental Biology, Disease Networks Research Unit, Faculty of Biochemistry and Molecular Medicine, Infotech Oulu, Kvantum Institute, Oulu University, Aapistie 5A, 90014 Oulu, Finland.

[e] iCell Group, Research and Development, Finnish Red Cross Blood Service, Haartmaninkatu 8, FIN-00290 Helsinki, Finland.

E-mail: sourov.chandra@aalto.fi, fabrizio.messina@unipa.it

Table S1 Atomic ratio of the metal atoms in the nanoclusters determined by atomic absorption spectroscopy (AAS).

Sample	Au : Ag	Au : Pd	Au : Pt
CNC-AuNC@Ag	20 : 1	-	-
CNC-AuNC@Pd	-	20 : 2	-
CNC-AuNC@Pt	-	-	20 : 2
CNC-AuNC@AgPd	20 : 1	20 : 2	-
CNC-AuNC@AgPt	20 : 1	-	20 : 3
CNC-AuNC@PdPt	-	16 : 2	16 : 3

Table S2 Elemental analysis by inductively coupled plasma mass spectrometry (ICP-MS)

Sample	Au (μg/L)	Ag (μg/L)	Pd (μg/L)	Pt (μg/L)	Atomic ratio
CNC-AuNC@AgPt	30500	1740	-	3290	Au : Ag : Pt ≈ 19 : 2 : 2
CNC-AuNC@AgPd	31800	2050	1990	-	Au : Ag : Pd ≈ 17 : 2 : 2

Table S3 Dynamic light scattering (DLS) and Zeta potential measurements of the CNCs, AuNCs and different CNC-NC nanocomposites

Sample	Zeta potential (mV)					Size (nm) measuring by DLS
	pH=2	pH=4	pH=7	pH=9	pH=11	
CNC	-25.1	-35.2	-46.2	-40.6	-27.7	158.4
AuNCs	3.2	-3.6	-3.5	-53.3	-53	1.5
CNC-AuNCs	-28.8	-29.8	-38.6	-43.2	-38.5	194.7
CNC-AuNCs@Ag	-30.6	-34.9	-35	-37.7	-37.7	194.7
CNC-AuNCs@AgPd	-34.3	-35.9	-40.1	-39.3	-30.5	194.3
CNC-AuNCs@AgPt	-30.8	-35.5	-40.1	-40.2	-37.8	179.9

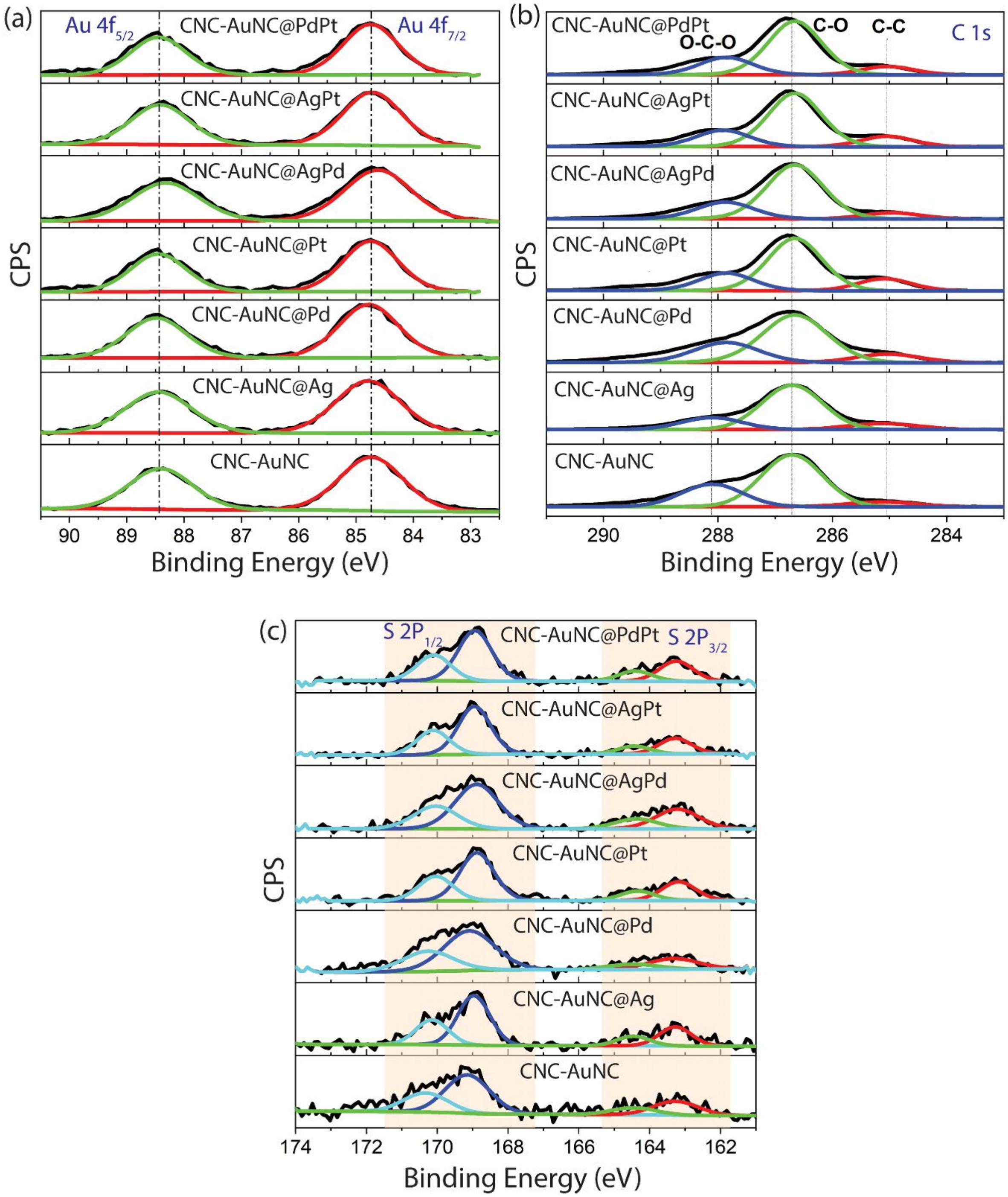

Figure S1 (a) Au 4f, (b) C 1s and (c) S 2p XPS spectra of heteroatom doped CNC-NCs.

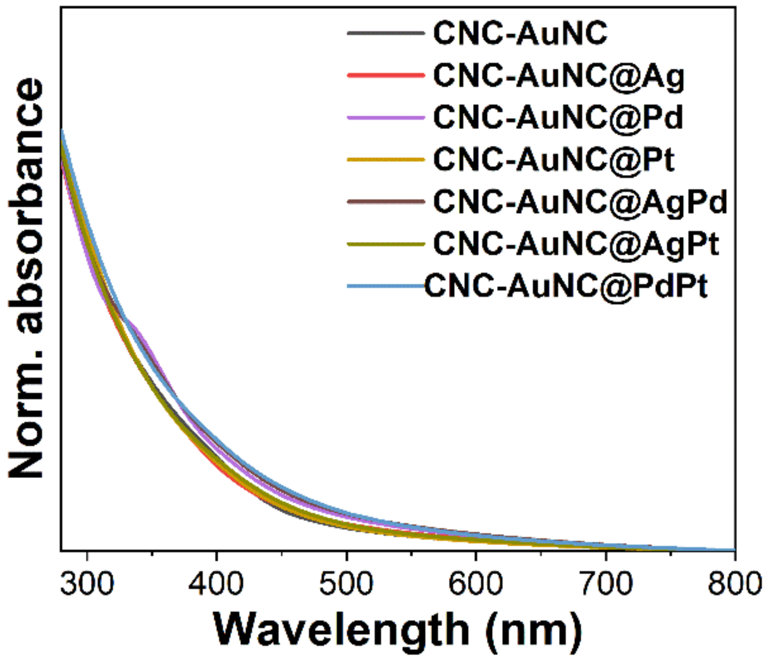

Figure S2 UV-vis absorption of the CNC-NCs composites.

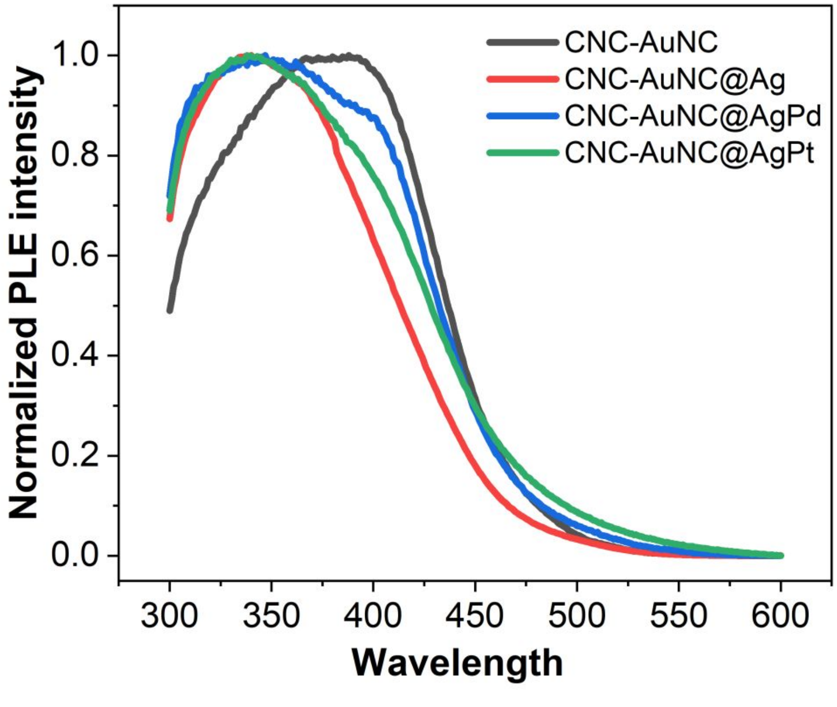

Figure S3 Photoluminescence excitation (PLE) spectra of the CNC-NCs.

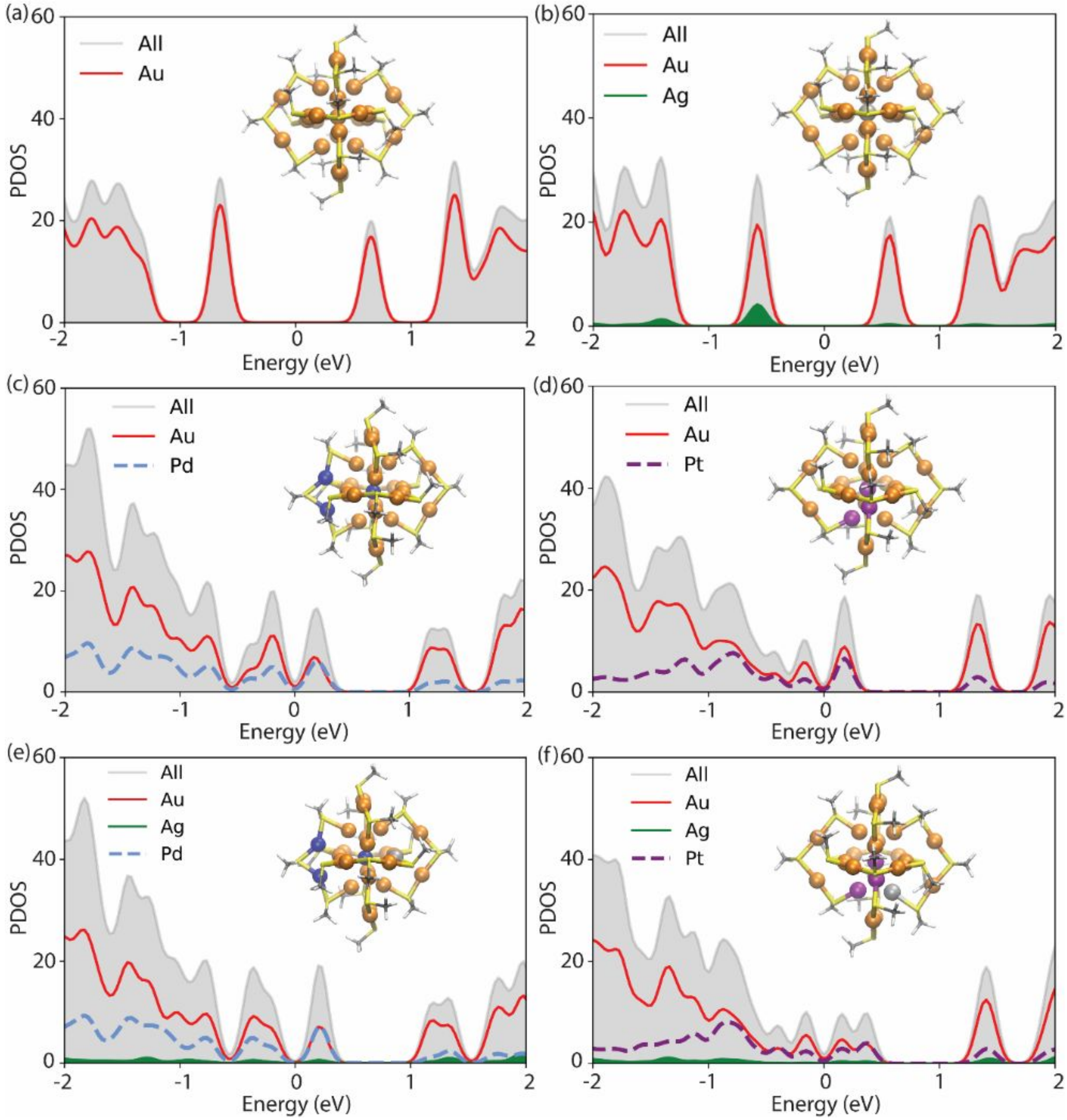

Figure S4 Projected density of states (PDOS) and corresponding atomic structure (inset) of the most stabilized nanoclusters (a) $[Au_{25}(SR)_{18}]^{-1}$, (b) $[Au_{24}Ag_{cc}(SR)_{18}]^{-1}$ (c) $[Au_{22}Pt_{cc+2oc}(SR)_{18}]$, (d) $[Au_{22}Pd_{cc+2sm}(SR)_{18}]$, (e) $[Au_{21}Ag_{1}Pt_{cc+2oc}(SR)_{18}]$ and (f) $[Au_{22}Ag_{1}Pd_{cc+2sm}(SR)_{18}]$. The PDOS plots in Figures S4 (a) and S4 (b) are adapted from ref 1.

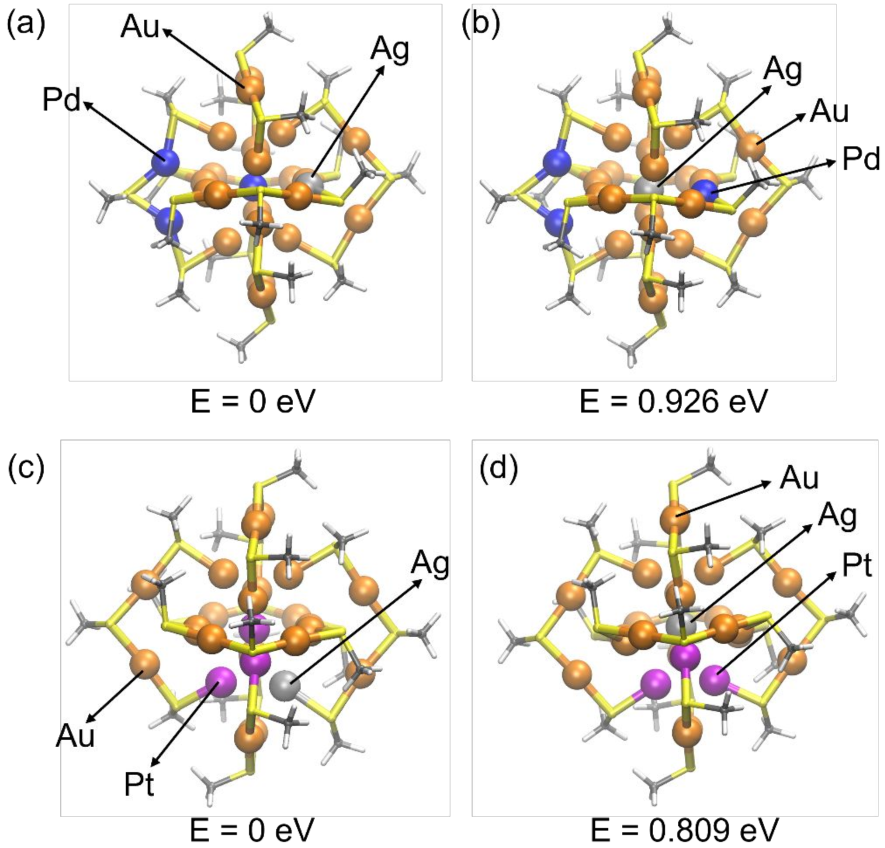

Figure S5 Predicted atomic structures of (a) $[Au_{22}Ag_{oc}Pd_{cc+2sm}(SR)_{18}]$, (b) $[Au_{22}Ag_{cc}Pd_{oc+2sm}(SR)_{18}]$, (c) $[Au_{21}Ag_{oc}Pt_{cc+2oc}(SR)_{18}]$ and (d) $[Au_{21}Ag_{cc}Pt_{3oc}(SR)_{18}]$ NCs (cc = central core, oc = outer core, and sm = staple motifs).

Table S4 The calculated HOMO-LUMO energy gap of the listed clusters.

Clusters	HOMO-LUMO gap
$[Au_{25}(SR)_{18}]^{-1}$	1.253 eV
$[Au_{24}Ag_{cc}(SR)_{18}]^{-1}$	1.099 eV
$[Au_{21}Ag_{1}Pt_{cc+2oc}(SR)_{18}]$	0.333eV
$[Au_{21}Ag_{1}Pd_{cc+2sm}(SR)_{18}]$	0.314eV
$[Au_{22}Pt_{cc+2oc}(SR)_{18}]$	0.307 eV
$[Au_{22}Pd_{cc+2sm}(SR)_{18}]$	0.403 eV

Table S5 Photoluminescence quantum yields (QY) of the different NCs over the CNC surfaces.

Sample	QY
CNC-AuNC	3.5%
CNC-AuNC@Ag	23%
CNC-AuNC@AgPd	3.9%
CNC-AuNC@AgPt	4.4%
CNC-AuNC@Pd	1.9%
CNC-AuNC@Pt	≤ 0.1%
CNC-AuNC@PdPt	≤ 0.1%

Table S6 Relative intensities and spectral positions of the Green (G) and Orange/Red (O/R) bands.

Sample	G band		O/R band	
	Relative intensity	Peak position	Relative intensity	Peak position
CNC-AuNCs	12%	500 nm	88%	630 nm
CNC-AuNC@Ag	15%	480 nm	85%	620 nm
CNC-AuNC@AgPd	33%	510 nm	67%	680 nm
CNC-AuNC@AgPt	13%	500 nm	83%	700 nm

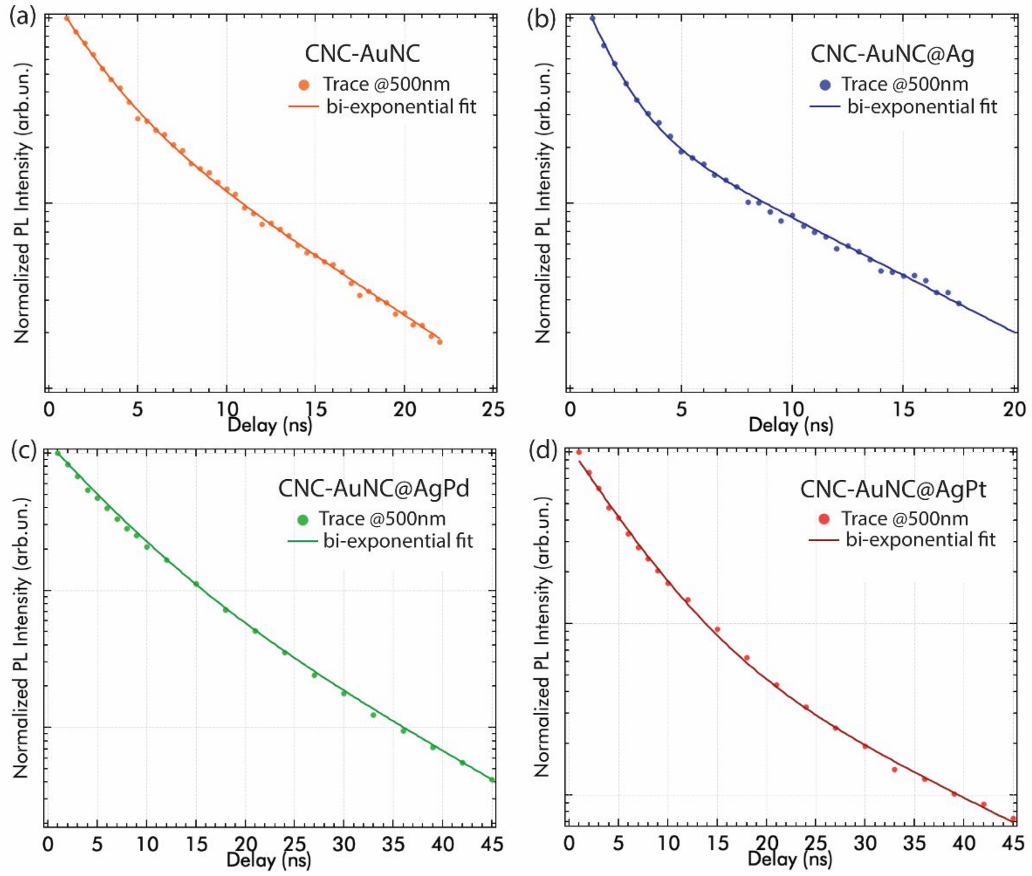

Figure S6 Decimal semilogarithmic plot of the decay traces of the green band and associated least-square fitting curves of (a) CNC-AuNC, (b) CNC-AuNC@Ag, (c) CNC-AuNC@AgPd and (d) CNC-AuNC@AgPt. The resulting fit parameters are enlisted in Table S5.

Table S7 Fit parameters for the decay kinetics of the green bands shown in Figure S5.

Sample	A_1(%)	τ_1(ns)	A_2(%)	τ_2(ns)
CNC-AuNC	61±4	2.2±0.2	39±5	6.9±0.2
CNC-AuNC@Ag	69±2	1.2±0.2	31±5	7.0±0.2
CNC-AuNC@AgPd	75±6	4.9±0.2	25±4	10.7±0.2
CNC-AuNC@AgPt	87±7	4.6±0.2	13±3	16.2±0.2

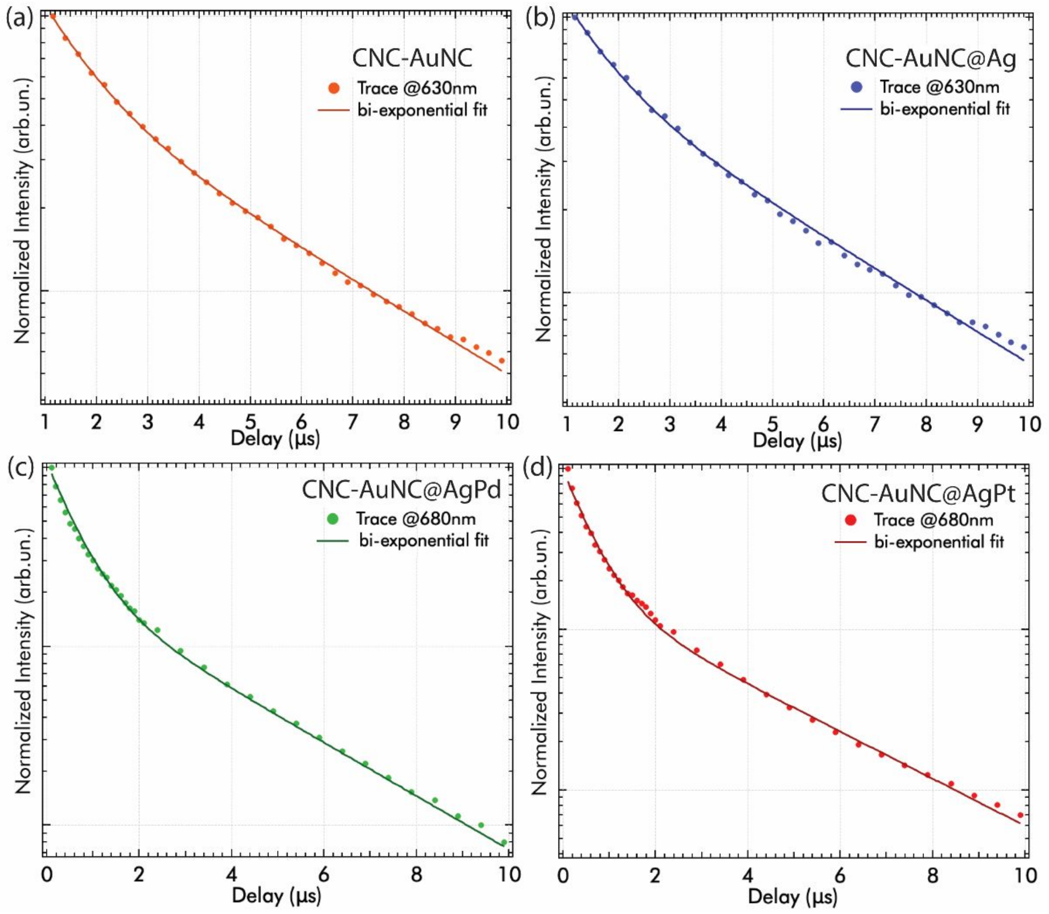

Figure S7 Decimal semilogarithmic plot of the decay traces of the orange/red band and associated least-square fitting curves of the (a) CNC-AuNC, (b) CNC-AuNC@Ag, (c) CNC-AuNC@AgPd and (d) CNC-AuNC@AgPt. The resulting fit parameters are enlisted in Table S6.

Table S8 Fit parameters for the decay kinetics of the orange/red bands shown in Figure S6.

Sample	A_1(%)	τ_1(µs)	A_2(%)	τ_2(µs)
CNC-AuNCs	48±3	0.9±0.2	52±4	3.8±0.2
CNC-AuNC@Ag	44±3	0.9±0.2	56±4	3.8±0.2
CNC-AuNC@AgPd	73±6	0.6±0.2	27±2	2.9±0.2
CNC-AuNC@AgPt	79±6	0.5±0.2	21±2	3.0±0.2

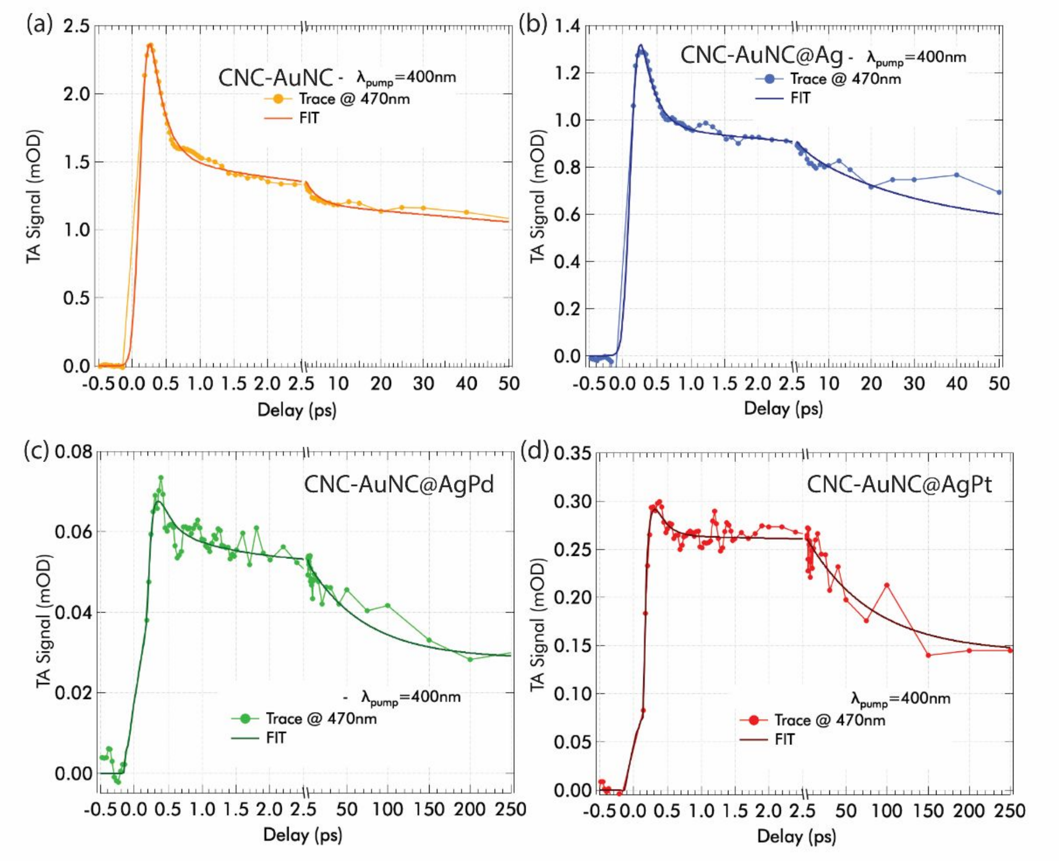

Figure S8 Fitted TA kinetic traces of the (a) CNC-AuNC (b) CNC-AuNC@Ag, (c) CNC-AuNC@AgPd and (d) CNC-AuNC@AgPt at 470nm. The resulting timescales are enlisted in Table S4.

Table S9 Parameters resulting from the fitting of the TA kinetics reported in Figure S7.

Sample	A_1(%)	τ_1'(ps)	A_2(%)	τ_2'(ps)	A_3(%)	τ_3'
CNC-AuNCs	55±4	0.13±0.04	10±2	2.9±0.1	35±3	≥1ns
CNC-AuNC@Ag	46±3	0.18±0.05	25±2	(3.1±0.1)+(30±2)	29±4	≥1ns
CNC-AuNC@AgPd	22±2	0.11±0.04	43±3	(1.9±0.1)+(79±5)	35±5	≥1ns
CNC-AuNC@AgPt	21±3	0.16±0.04	46±3	81±5	43±4	≥1ns

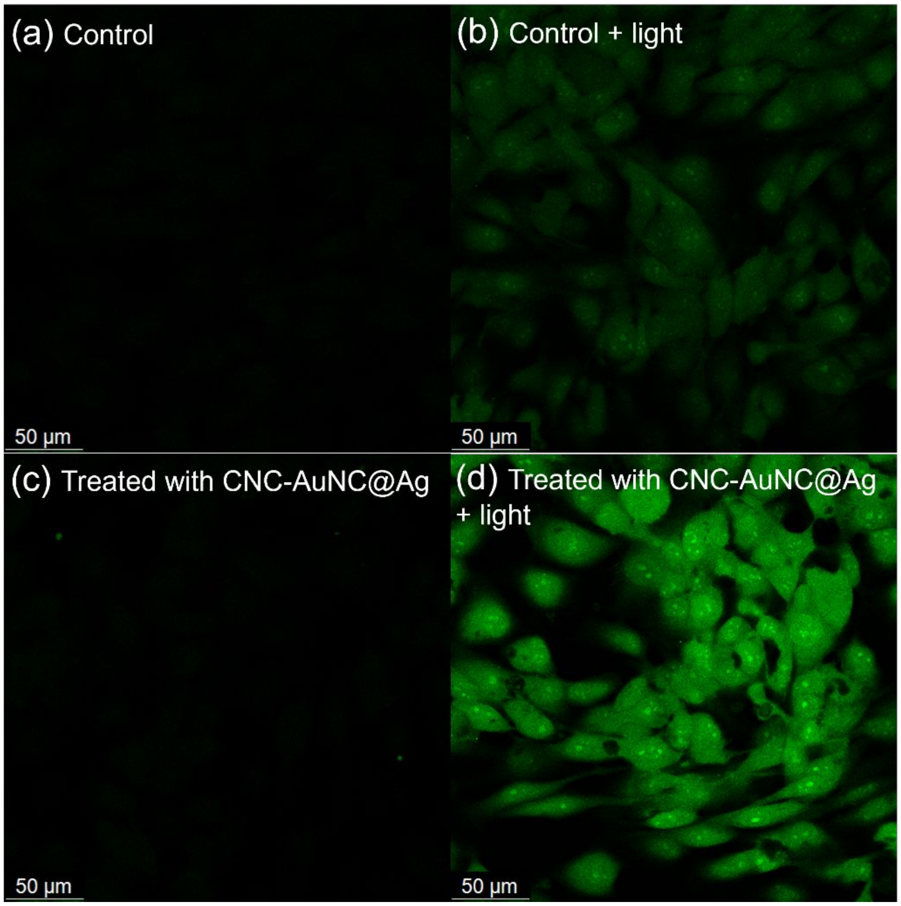

Figure S9 Confocal microscopy images (λ_{ex} = 485 nm) of 786-O cells: (a, b) control and (c, d) incubated with CNC-AuNC@Ag. ROS production was visualized by using DCFH-DA assay. Images were taken either before light exposure (a, c) or immediately after light exposure (blue light, 405 nm) for 30 min (b, d).

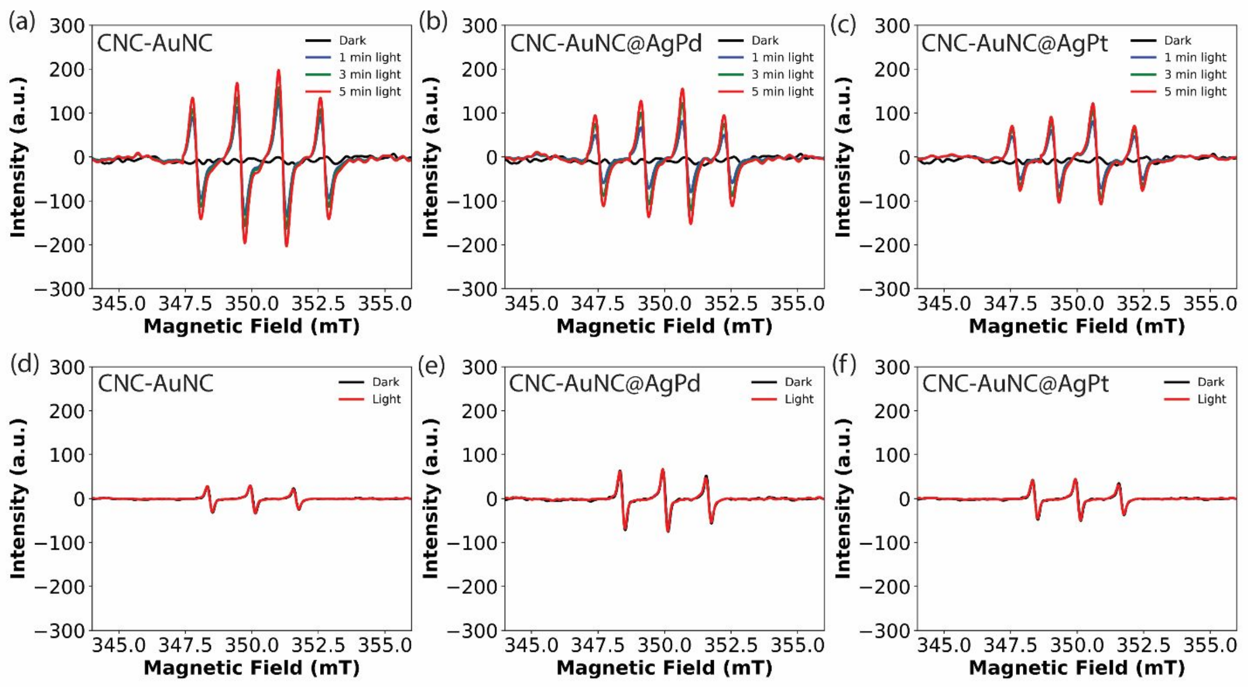

Figure S10 ESR spectra of DMPO-OOH adducts generated by (a) CNC-AuNC, (b) CNC-AuNC@AgPd and (c) CNC-AuNC@AgPt in aqueous solutions containing 1M DMPO with different light irradiation times. ESR spectra of singlet oxygen adducts generated by (d) CNC-AuNC, (e) CNC-AuNC@AgPd and (f) CNC-AuNC@AgPt in aqueous solutions containing 1M TEMP with different light irradiation times.

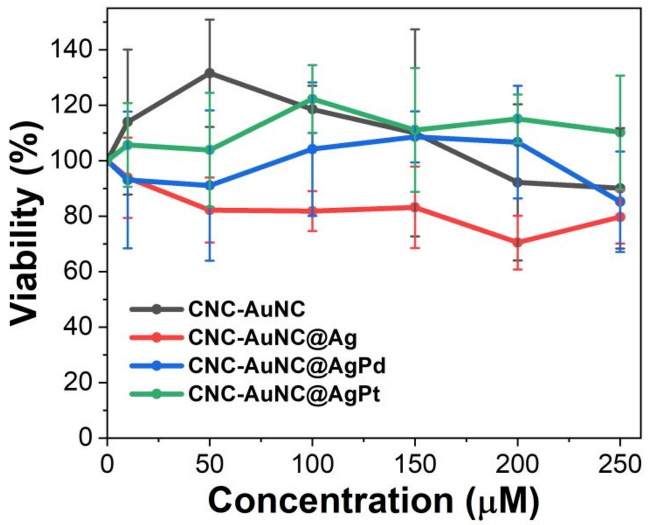

Figure S11 Cell viability of NIH3T3 cells using CCK-8 assay (n $\geq$ 4) incubated for 24 hours with undoped CNC-AuNC and metal doped CNC-NC samples.

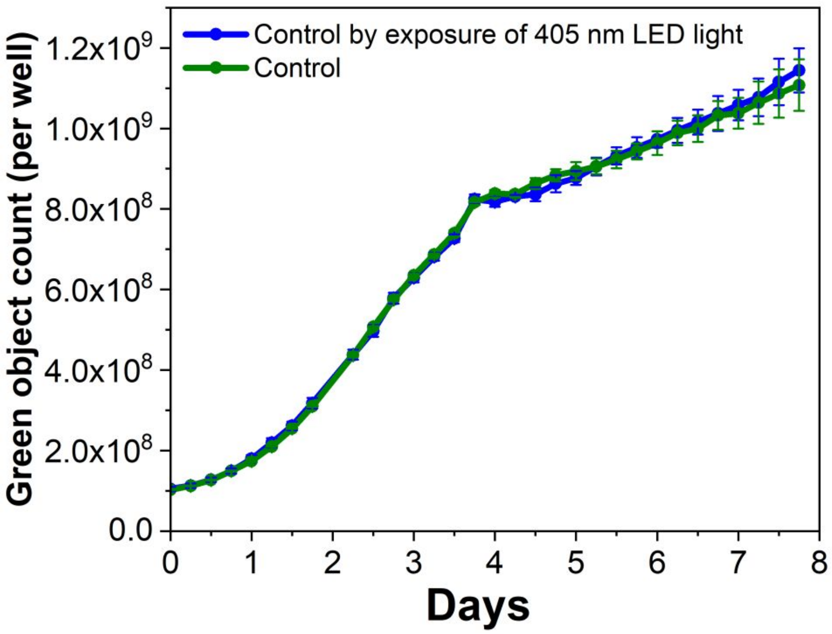

Figure S12 The growth curves of 786-O PG cells in absence (control) and under exposure of blue LED light (λ_{ex} = 405 nm).

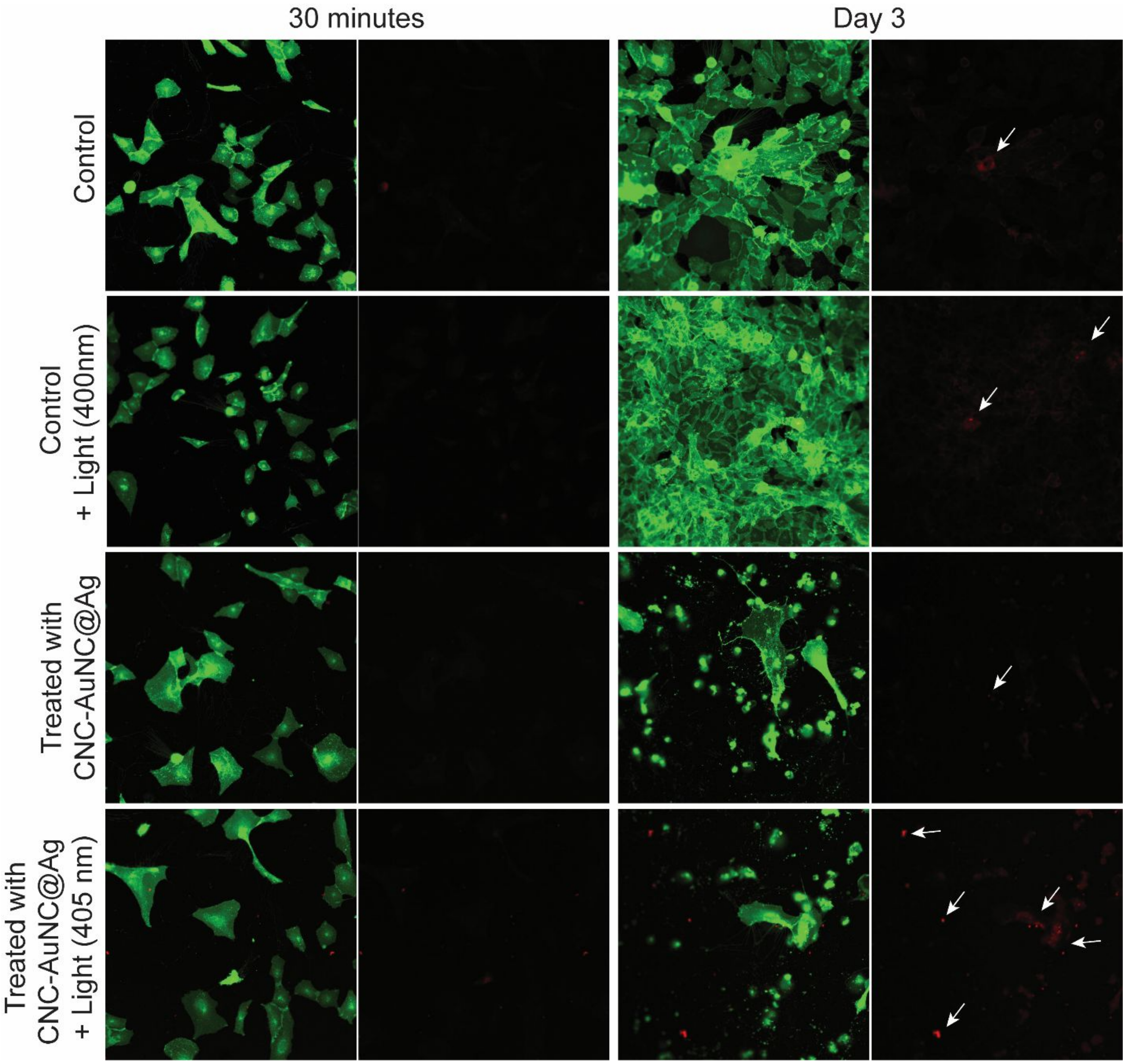

Figure S13 Confocal microscopic images of SH-SY5Y cells: control, exposed to light, treated with CNC-AuNC@Ag and treated with CNC-AuNC@Ag + exposed to light for 30 minutes and 3 days after light exposure. Representative images taken in green (GFP) and red (Annexin V) channels are shown, Annexin V-positive cells are marked with arrows.

Supplementary Video S1. A general view of chicken embryo *ex ovo* culture 4 days after transplantation of membrane rings with SH-SY5Y neuroblastoma spheroids. A head of chicken embryo, CAM, and vascularized spheroids (both in brightfield and green fluorescence channel) are visible.

Supplementary Video S2. A general view of chicken embryo *ex ovo* culture 4 days after transplantation of membrane rings with SH-SY5Y neuroblastoma spheroids and 3 days after addition of CNC-AuNC@Ag. A head of chicken embryo, CAM, and vascularized spheroids covered with CNC (both in brightfield and green fluorescence channel) are visible.

References:

(1) Chandra, S.; Sciortino, A.; Shandilya, S.; Fang, L.; Chen, X.; Nonappa; Jiang, H.; Johansson, L. S.; Cannas, M.; Ruokolainen, J.; Ras, R. H. A.; Messina, F.; Peng, B.; Ikkala, O. Core-Selective Silver-Doping of Gold Nanoclusters by Surface-Bound Sulphates on Colloidal Templates: From Synthetic Mechanism to Relaxation Dynamics. *Adv Opt Mater* **2023**, *11* (1), 2201901. https://doi.org/10.1002/adom.202201901.
